# Amicoumacins produced by the native citrus microbiome isolate *Bacillus safensis* inhibit the Huanglongbing-associated bacterial pathogen “*Candidatus* Liberibacter asiaticus”

**DOI:** 10.1128/aem.00869-25

**Published:** 2025-07-31

**Authors:** Flávia C. Vieira, Kranthi K. Mandadi, Manikandan Ramasamy, Amancio de Souza, Kiana Callahan, Corrie Fyle, Andrew Kamemoto, Amanda G. Koontz, Christopher Yang, Robert Crowley, Kevin G. M. Kou, Katherine N. Maloney, M. Caroline Roper

**Affiliations:** 1 Department of Microbiology and Plant Pathology, University of California 207030, Riverside, California, USA; 2 Texas A&M AgriLife Research and Extension Center, Texas A&M University System 2655, Weslaco, Texas, USA; 3 Department of Plant Pathology and Microbiology, Texas A&M University 199053, College Station, Texas, USA; 4 Institute for Advancing Health Through Agriculture, Texas A&M AgriLife 117324 , Weslaco, Texas, USA; 5 Institute for Integrative Genome Biology, University of California 117242, Riverside, California, USA; 6 Department of Chemistry, Point Loma Nazarene University 7116, San Diego, California, USA; 7 Department of Chemistry, University of California 335758 , Riverside, California, USA; The University of Tennessee Knoxville, Knoxville, Tennessee, USA

**Keywords:** citrus greening, citriculture, natural products, antimicrobials

## Abstract

Huanglongbing (HLB) is a devastating citrus disease associated with the gram-negative, phloem-limited, and unculturable bacterium “*Candidatus* Liberibacter asiaticus (*C*Las),” which is transmitted by the Asian citrus psyllid *Diaphorina citri*. Despite extensive research, effective, long-term, and sustainable solutions for managing HLB remain elusive. Oxytetracycline (OTC) is currently used as an emergency measure, but there is an urgent need for alternative compounds to complement or replace OTC. In this study, we identified amicoumacins, a class of antimicrobial compounds produced by the bacterium *Bacillus safensis* CB729 isolated from the citrus microbiome, and demonstrated their ability to suppress *C*Las. Genome mining of *B. safensis* CB729, combined with metabolomic analysis and bioassay-guided fractionation, revealed the presence of amicoumacins and related derivatives in fractions inhibitory to *Liberibacter crescens*, a culturable surrogate for *C*Las. We tested commercially available synthetic amicoumacins A and B, along with a *B. safensis*-derived amicoumacin mixture, against *L. crescens* and *C*Las. We determined the MICs of amicoumacin A (1.25 µg/mL) and amicoumacin B (10 µg/mL) against *L. crescens*. Furthermore, amicoumacin B and the amicoumacin mixture significantly reduced *C*Las populations in *ex vivo* citrus hairy root assays. This study highlights the potential of amicoumacins as a promising group of natural products for the management of HLB, offering valuable insights for the development of novel and sustainable disease control strategies.

**IMPORTANCE:**

For two decades, the citrus industry has been severely impacted by Huanglongbing (HLB), a devastating disease caused by “*Candidatus* Liberibacter asiaticus (*C*Las)” and transmitted by the Asian citrus psyllid (*Diaphorina citri*). Despite extensive research, effective, long-term, and sustainable solutions remain unavailable for growers. Currently, medically relevant antibiotics, such as oxytetracycline (OTC), are used as an emergency response to combat HLB in Florida, the most affected citrus-producing state in the U.S. This underscores the urgent need for alternative treatments that can be used in rotation or as replacements for OTC. Here, we present amicoumacins, a group of bioactive secondary metabolites with antibiotic properties. We identified amicoumacin B and its derivatives from the culture broth of a *Bacillus safensis* isolate, native to citrus, and demonstrated their ability to inhibit *Liberibacter* spp. and reduce *C*Las populations in citrus tissue. This study highlights how microbial discovery can lead to the identification of antimicrobial compounds with potential applications in plant disease management.

## INTRODUCTION

Citrus is a major subtropical and tropical fruit crop with high nutritional and economic value, largely cultivated in China, Brazil, the United States, the European Union, Mexico, and Egypt (1, 2). The current global orange production is forecast to be 45.2 million tons (2). Nevertheless, citrus production and sustainability have been impacted and threatened worldwide by Huanglongbing (HLB), associated with the unculturable phloem-limited bacteria, “*Candidatus Liberibacter* spp.” (3). In the U.S., the prevalent associated pathogen is “*Candidatus Liberibacter* asiaticus” (*C*Las), which is vectored by the Asian citrus psyllid, *Diaphorina citri* (3, 4). HLB causes severe disruption of photosynthate transport due to phloem blockage mediated by the deposition of callose in the phloem cells (5). This disruption results in the typical HLB symptoms that include accumulation of starch in the leaves, yellow shoots, thinning of the canopy, premature fruit drop, reduced fruit quality, root mass loss, and significant yield reduction, all of which eventually lead to tree death (6, 7). HLB has been present in the U.S. for two decades. It was first detected in Florida in 2005 (3) and became endemic in 2013. HLB has since drastically altered the landscape of the U.S. citrus industry by causing a significant reduction in citrus acreage and yield (8, 9).

Significant progress has been made in developing sustainable solutions for HLB, including the development of HLB-resistant scion and rootstock citrus varieties (10–12), nutritional enhancement programs, and the use of hormones and plant-immunity inducers (13–15). Additionally, novel chemistries for vector control (16) and antimicrobial compounds against *C*Las have been explored (17–22). While some of these potential solutions have undergone the regulatory approval process to become available to growers, investigations into additional long-term and sustainable solutions are necessary. Currently, the use of antibiotics to combat HLB has increased in Florida as an emergency response. Initially, oxytetracycline (OTC) and streptomycin were approved for foliar application on HLB-affected trees. Trunk injection is the most effective method for delivering these compounds directly to the tree’s vascular system (23–26). In 2022, Florida approved the commercial application of OTC via trunk injection, and it has since been widely implemented. This cultural practice enhances fruit yield and quality and improves overall tree health (23, 25, 26). Despite the immediate effectiveness of OTC in managing HLB, there is growing concern among scientists and growers about the potential development of antibiotic-resistant *C*Las in citrus orchards under high HLB pressure. This underscores the urgent need to identify alternative compounds that can be used in rotation with or as substitutes for OTC for medium- to long-term management of HLB (27).

Plant microbiomes play a crucial role in enhancing plant health, productivity, and disease resistance (28). Specifically, constituents within the microbiome can produce a unique array of bioactive compounds that can benefit the plant host and can be a valuable resource for bioprospecting of microbially produced antimicrobial natural products. In a prior bioprospecting effort, we generated a citrus-associated microbial collection from groves under high (Florida) and low (California) HLB pressure (29). Notably, *Bacillus* species were the most abundant isolates in our citrus microbial culture collection, constituting 37.4% of isolates from leaves, 34.7% of isolates from stems, and 28.5% of isolates from roots (29).

*Bacillus* spp. are commonly found in plant-associated microbiomes, including soil, the rhizosphere, and the plant endosphere (30–32) and can produce an array of secondary metabolites with antibacterial and antifungal properties (33). Additionally, *Bacillus* spp. compete for niches and nutrients with pathogens and can induce systemic resistance in plants (34). As a result, several commercially available products have emerged based on beneficial strains of *Bacillus*, such as *B. velezensis*, *B. amyloliquefaciens*, *B. subtilis*, *B. pumilus*, *B. cereus*, *B. megaterium*, and *B. licheniformis* (32, 33, 35, 36). Among the bioactive secondary metabolites of some *Bacillus* strains, amicoumacins are a small group of compounds that have drawn attention in the last decade for their bioactivity (37).

Through mining our citrus-associated microbial collection for isolates with anti-*Liberibacter* properties, using the surrogate species for *C*Las, *Liberibacter crescens* (38), we identified a *Bacillus* isolate (designated strain CB729) as particularly effective in inhibiting *L. crescens* in bioactive fractions. Here, we describe the identification and isolation of amicoumacins produced by *Bacillus safensis* CB729 and their *ex vivo* suppression of the HLB-associated pathogen, *C*Las, in citrus tissue.

## RESULTS

### *B. safensis* CB729 harbors the amicoumacin biosynthetic gene cluster

**Fig 1:**
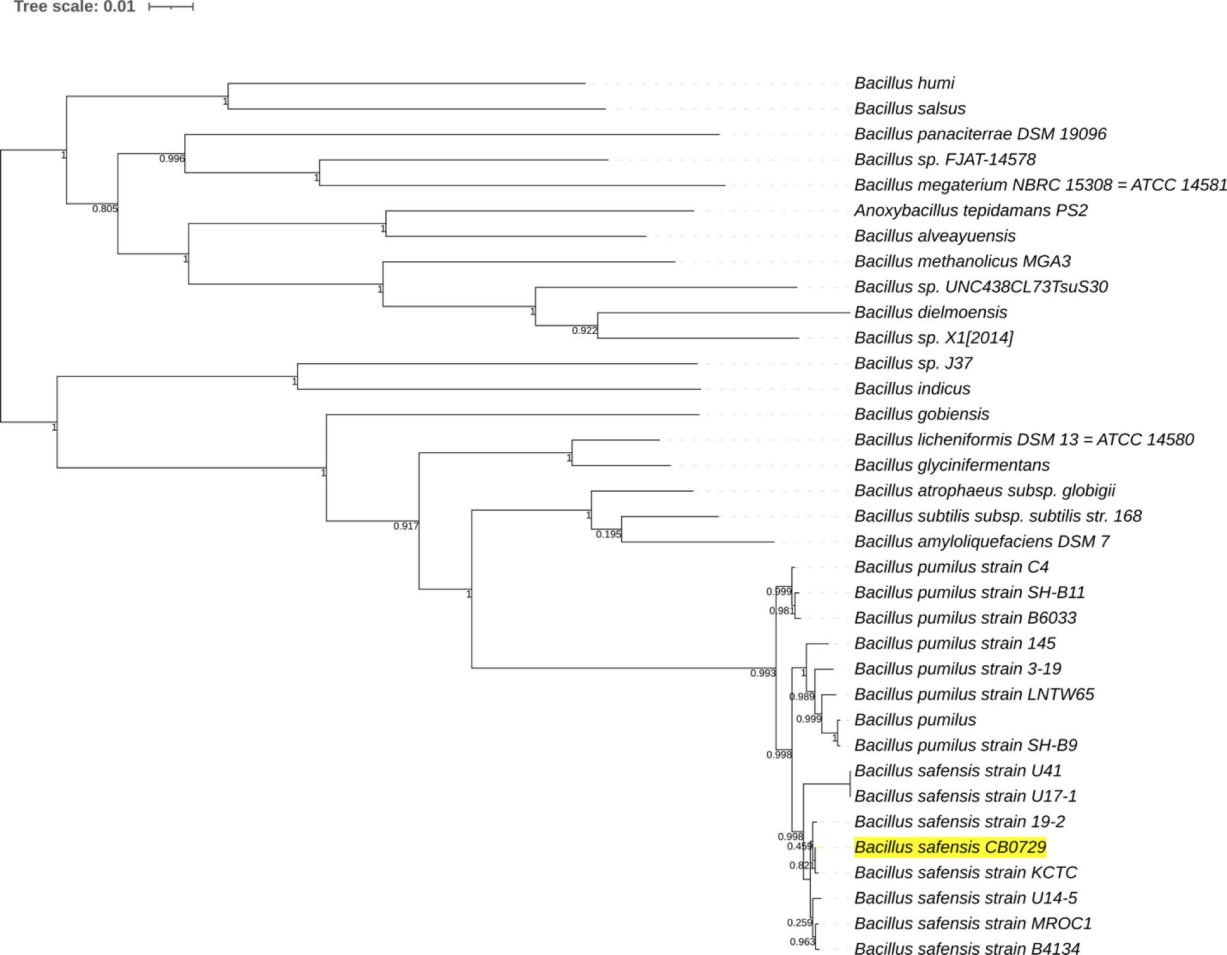
Phylogenetic tree of *Bacillus*. The phylogenetic tree was constructed using the SpeciesTree (v2.2.0) app of Kbase, using a set of 49 core, universal genes defined by COG (Clusters of Orthologous Groups) gene families. *B. safensis* CB729 is highlighted in yellow.

We initiated the screening of the citrus-associated microbial collection by assessing the *in vitro* antagonistic activity of various microbial isolates against *L. crescens* (29). From a subset of 10 isolates that demonstrated inhibitory activity against *L. crescens*, isolate CB729 exhibited particularly strong inhibition (Fig. S1), leading to its selection for further investigation. Whole-genome sequencing confirmed that isolate CB729 belongs to the *B. safensis* species, and its phylogenetic placement is illustrated in the species tree, compared to several other *B. safensis* strains and to closely related species, *B. pumilus* strains (Fig. 1). The draft genome assembly of *B. safensis* CB729 (GCA_036621555.1) is described in detail in reference 39. In summary, the genome assembly resulted in 22 contigs, accounting for a total size of 3,677,118 bp, N50 of 871,191 bp, and GC content of 41.50%. Genome annotation analysis identified 3,763 predicted genes and 3,654 protein-coding sequences (CDS), and taxonomy was confirmed with 97.4% of average nucleotide identity (ANI; Table S1; Fig. S2).

**Fig 2:**
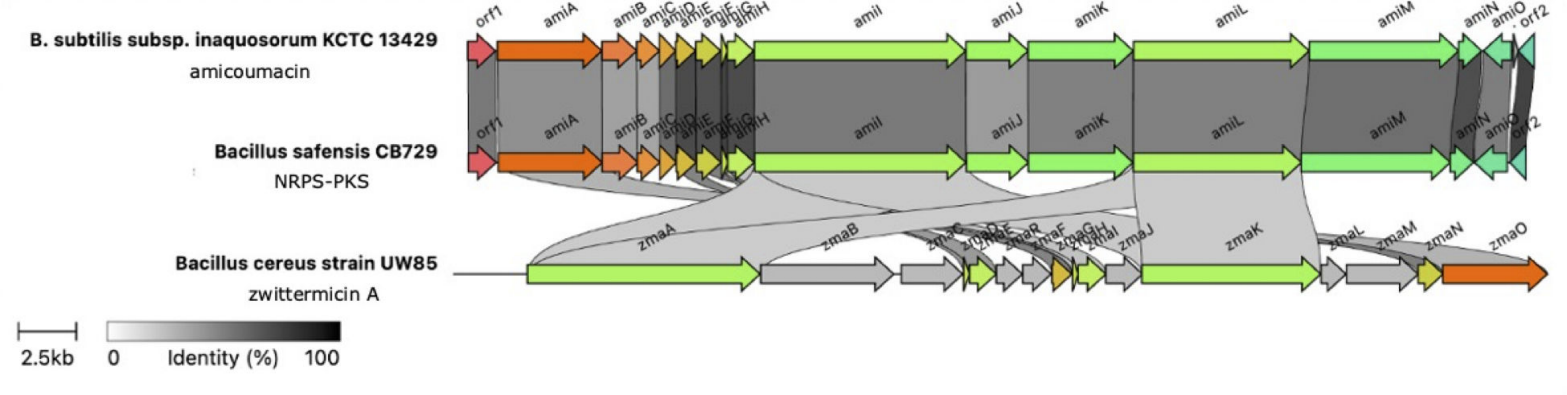
Gene cluster comparison of NRPS-PKS of *B. safensis* CB729 to the characterized amicoumacin BGC of *B. subtilis* subsp. *inaquosorum* KCTC 13429 and the zwittermicin A (ZmA) BGC of *Bacillus cereus* strain UW85. This analysis was performed using Clinker on CAGECAT. Genes are colored based on homology, and gray-colored genes have no similarity with other genes in the alignment. The percentage of identity between genes is represented in shades of gray.

Predicted secondary metabolite biosynthetic gene cluster (BGC) analysis in antiSMASH revealed 11 BGCs with annotations to known clusters in the Minimum Information about a Biosynthetic Gene Cluster (MIBiG) database, including the bacillibactin, fengycin, bottromycin, zwittermicin A, lichenysin, bacilysin, and schizokinen clusters (Table 1). One hybrid non-ribosomal peptide synthase-polyketide synthase domain (NRPS-PKS) was identified in the genome of CB729 and was identified as similar to the zwittermicin A (ZmA) cluster of *B. cereus*. Subsequently, gene sequence alignment of the NRPS-PKS cluster to the characterized amicoumacin cluster of *B. subtilis* subsp. *inaquosorum* KCTC 13429 (40, 41) was performed. The analysis revealed a moderate identity (46%–80%) and a strong similarity (86%–100%) between clusters (Fig. 2), corroborating findings by reference 42. Table 2 presents the proteins encoded by the NRPS-PKS cluster of *B. safensis* CB729, along with their proposed functions and their similarities to the characterized amicoumacin gene cluster in *B. subtilis* subsp. *inaquosorum* KCTC 13429, suggesting that the amicoumacin gene cluster is present in CB729.

**TABLE 1:** Predicted secondary metabolite gene clusters of *B. safensis* CB729^*a*^

Cluster	Type	Location	Size (nt)	Similar known cluster	Gene number
1	NRPS	8845–92557	83,713	Lichenysin	40
2	NRPS	25921–77649	51,729	Bacillibactin	45
3	Betalactone	28232–60483	32,252	Bottromycin A2	28
4	NRPS, T1PKS	63417–144369	80,953	Zwittermicin A	44
5	RiPP-like	189667–199993	10,327	–	14
6	Type 3 PKS	509215–550315	41,101	–	45
7	Terpene	588516–610393	21,878	–	19
8	Other	630481–671902	41,422	Bacilysin	46
9	Betalactone	680326–708735	28,410	Fengycin	24
10	NI-siderophere, terpene	1422699–1460346	37,648	Schizokinen	35
11	RRE-containing	1607651–1628556	20,906	–	20

^*a*^ – indicates that there was no match between the cluster sequence and any known cluster in the database.

**TABLE 2:** Proposed function and size of proteins encoded by the amicoumacin cluster of *B. safensis* CB729 and adjacent open reading frames and identity/similarity to the characterized amicoumacin gene cluster of *B. subtilis* subsp. *inaquosorum* KCTC 13429^*a*^

Protein	Size (aa)	Proposed function	Identity/similarity (%)	Accession no.
Orf1	399	MFS transporter (*Bacillus*)	59/100	WP_003240131.1
AmiA	1,489	Non-ribosomal peptide synthetase (*Bacillus inaquosorum*)	52/99	WP_003240128.1
AmiB	513	Serine hydrolase domain-containing protein (*Bacillus inaquosorum*)	48/93	WP_003240126.1
AmiC	326	Hypothetical protein (*Bacillus inaquosorum*)	46/97	WP_003240124.1
AmiD	236	Alpha/beta fold hydrolase (*Bacillus inaquosorum*)	65/99	WP_003240123.1
AmiE	285	3-Hydroxyacyl-CoA dehydrogenase family protein (*Bacillus*)	77/99	WP_003240121.1
AmiF	350	HAD-IIIC family phosphatase (*Bacillus inaquosorum*)	76/99	WP_003240119.1
AmiG	90	Acyl carrier protein (*Bacillus inaquosorum*)	80/98	WP_268315241.1
AmiH	380	Acyl-CoA dehydrogenase family protein (*Bacillus inaquosorum*)	77/99	WP_003240115.1
AmiI	3,029	Hybrid non-ribosomal peptide synthetase/type I polyketide synthase (*Bacillus inaquosorum*)	60/100	WP_003240114.1
AmiJ	889	Non-ribosomal peptide synthetase (*Bacillus inaquosorum*)	48/100	WP_003240112.1
AmiK	1,500	Type I polyketide synthase (*Bacillus inaquosorum*)	57/100	WP_003240111.1
AmiL	2,410	Type I polyketide synthase (*Bacillus inaquosorum*)	58/99	WP_003240108.1
AmiM	2,136	Type I polyketide synthase (*Bacillus inaquosorum*)	64/99	WP_003240106.1
AmiN	335	Phosphotransferase enzyme family protein (*Bacillus*)	76/99	WP_003240104.1
AmiO	468	Alkaline phosphatase (*Bacillus inaquosorum*)	67/86	WP_003240102.1
Orf2	230	MgtC/SapB family protein (*Bacillus inaquosorum*)	80/100	WP_003240098.1

^*a*^ MFS, major facilitator superfamily.

### Anti-*Liberibacter* fractions of *B. safensis* CB729 contain amicoumacins

**Fig 3:**
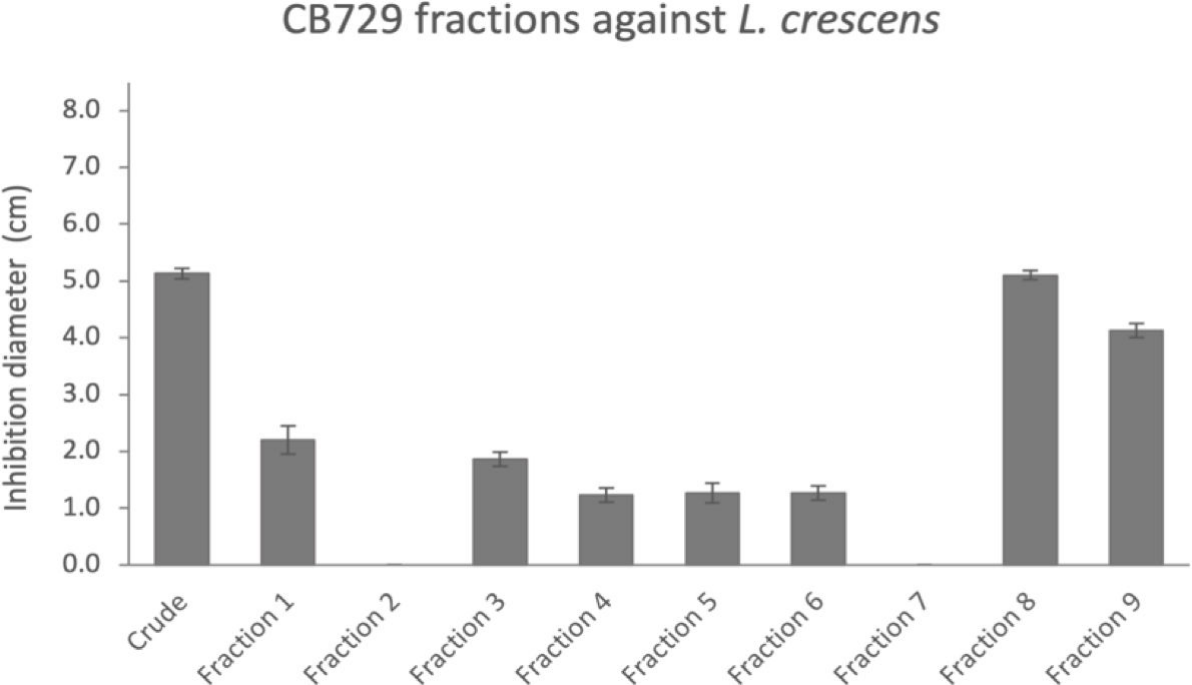
*In vitro* inhibition bioassay of CB729 fractions against *L. crescens*. Initial fractionation of the crude extract of CB729 resulted in nine fractions, seven of which were inhibitory to *L. crescens. N*-acetyl-amicoumacin C was identified in subsequent reversed-phase HPLC of fraction 6. Each fraction was tested in triplicate.

Initial fractionation of the crude extract of CB729 by normal phase medium-pressure liquid chromatography resulted in nine fractions, seven of which were inhibitory to *L. crescens* (Fig. 3). Liquid chromatography-mass spectrometry (LC-MS) analysis revealed a family of compounds with similar masses around *m/z* 400–500 and a distinctive UV chromophore in many of the fractions. Reversed-phase high-performance liquid chromatography (HPLC) of fraction six gave four subfractions (6a–6d), one of which (fraction 6d) was identified as pure *N*-acetylamicoumacin C based on an [M-H]^−^ peak in the LC-MS at *m/z* 447.1, and comparison of nuclear magnetic resonance (NMR) data with those previously reported (43) (Table S2; Fig. S3 to S6). Although the isolated *N*-acetylamicoumacin C was inactive against *L. crescens*, its structural relationship to known antibiotic amicoumacins led us to explore the extract further. In the process of isolating amicoumacins, fractionation of CB729 crude extract was performed at least seven times, and amicoumacins were consistently detected in the fractions that were inhibitory to *L. crescens* for all seven fractionation runs (Table S3). The experiment was repeated with seven biological replicates, each with at least three technical replicates per fraction.

### Molecular networking reveals amicoumacins in crude extracts of *B. safensis* CB729

**Fig 4:**
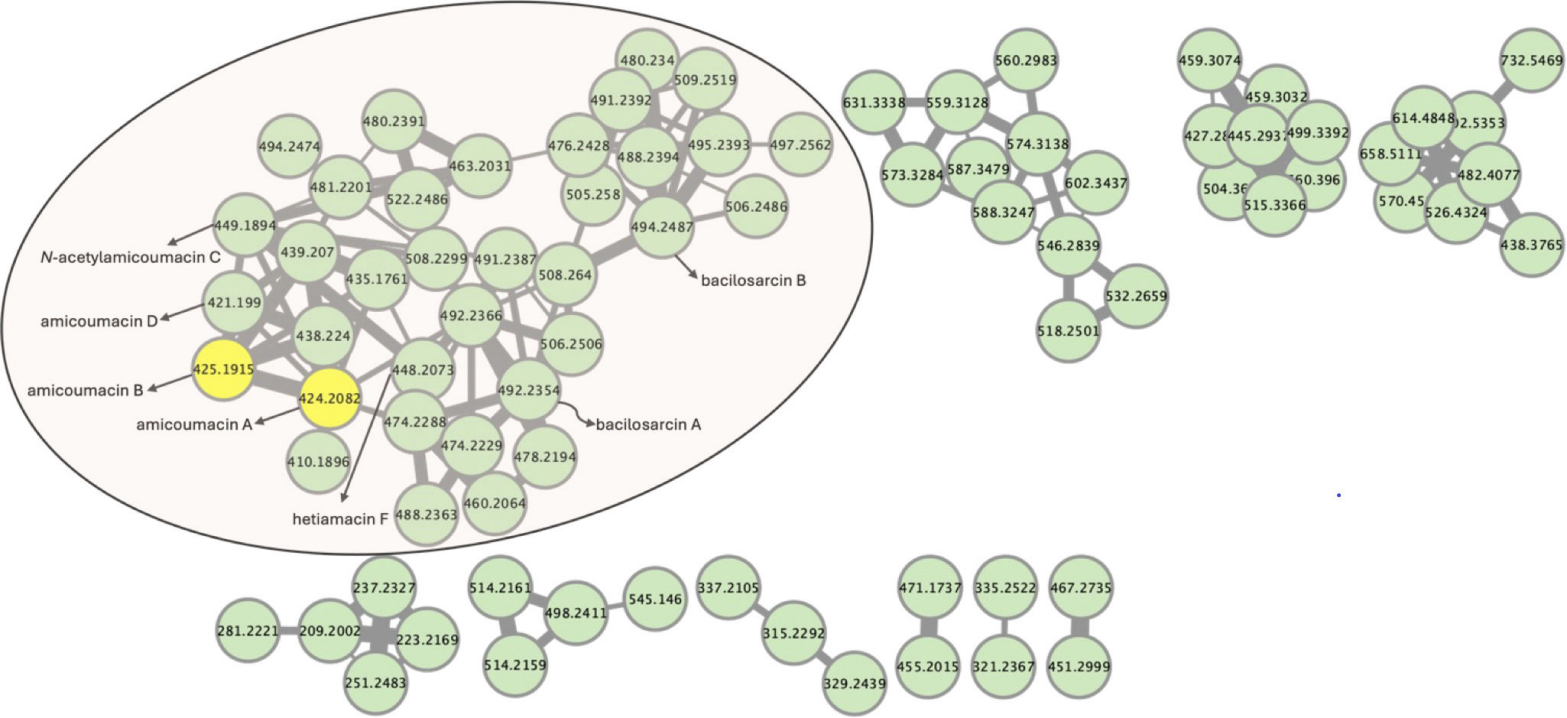
FBMN analysis of CB729 crude extract. FBMN generated in GNPS2 and visualized in Cytoscape v.3.9.1. Metabolites produced by CB729 in SYC media were clustered in molecular families, based on MS/MS spectra similarity. *m/z* of the precursor ion is annotated inside each node, and edge widths are scaled based on cosine score (a measure of spectral similarity). Synthetic amicoumacins A and B were included as standards and are highlighted in yellow. The large molecular family highlighted in a circle contains the amicoumacins. Known compounds are labeled based on comparison with literature MS/MS spectra.

To explore the full metabolite profile of the CB729 extract, we performed a feature-based molecular networking (FBMN) analysis of the CB729 crude extract. Molecular networking is a tool that permits the rapid annotation of known metabolites, or families of related metabolites, based on their fragmentation mass spectra (MS/MS). In the obtained FBMN, 20,656 individual MS/MS spectra were organized into 655 nodes and 1,174 edges (Fig. S7). The network was then filtered to remove nodes corresponding to compounds observed in the SYC media blank and LC-MS system blank, thereby reducing the size of the network to 80 nodes and 159 edges (Fig. 4). The inclusion of MS/MS data of synthetic amicoumacins A and B as standards in the FBMN analysis aided in the identification of an amicoumacin molecular family. The amicoumacin molecular family consisted of 35 nodes and 83 edges, ranging from *m/z* 410.1896 to 522.2486. Based on the molecular formulas predicted from the precursor ion *m/z* values and MS/MS fragmentation pattern, seven amicoumacin-related compounds were identified, including amicoumacins A, B, and D, *N*-acetylamicoumacin C, hetiamacin F, and bacilosarcins A and B (Table 3).

**TABLE 3:** Annotated compound information from FBMN analysis

Compound name	m/z	Base peak	Retention time	Cluster index (GNPS2)
Amicoumacin A	424.2082	250.1436	4.11	1,764
Amicoumacin B	425.1915	250.1434	4.22	2,070
Amicoumacin D	421.1990	250.1430	5.38	4,941
Hetiamacin F	448.2073	390.1538	4.24	2,010
*N*-acetylamicoumacin C	449.1894	449.1917	5.53	5,283
Bacilosarcin A	492.2354	474.2235	4.49	2,637
Bacilosarcin B	494.2487	494.2499	4.25	1,852

### Scaled-up production and purification of amicoumacin A

**Fig 5:**
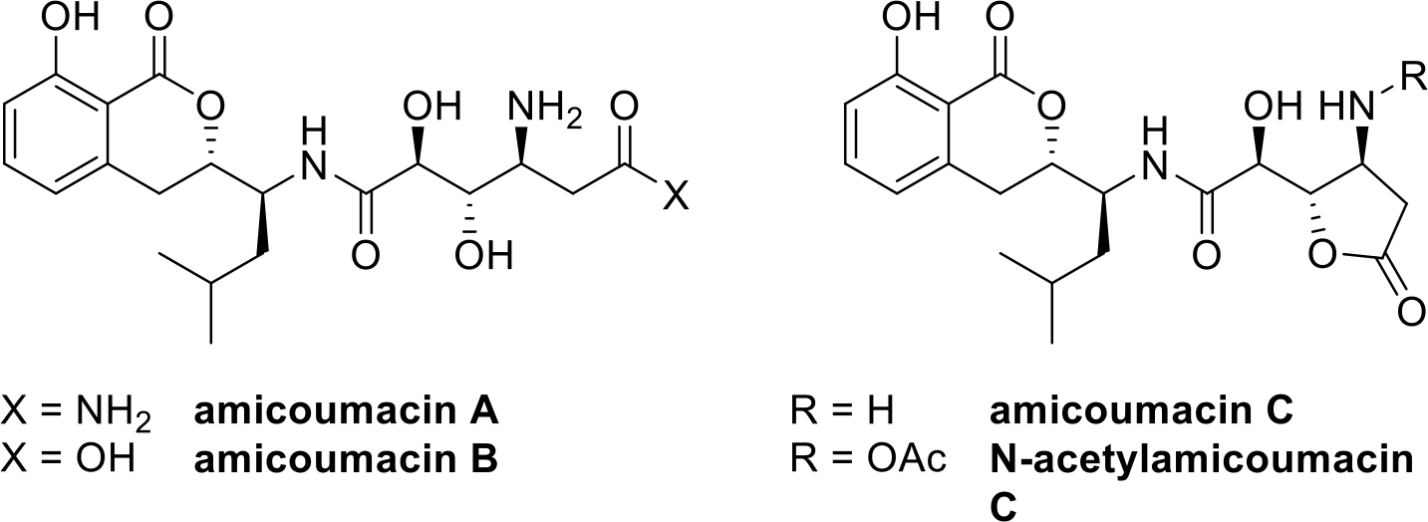
Structures of amicoumacins isolated from *B. safensis* CB729 broth in this work, resolved by LC-MS and NMR analysis.

Following the detection of amicoumacins in the crude extract of CB729, the bacterial fermentation conditions were optimized using SYC medium (44) for isolation of amicoumacin A, recognized in the literature to be the most bioactive of the amicoumacins (37, 43). SYC contains mainly sucrose, yeast extract, and calcium carbonate, and it has been associated with the improvement of amicoumacin production, in combination with abundant aeration and shorter cultivation periods (44). The *N*-acetylation of amicoumacin A during fermentation has been described as a strategy employed by the producer bacteria to reduce its toxicity (41, 43). To prevent this, we grew CB729 in 12 L of SYC in the presence of HP-20 solid-phase extraction resin to absorb amicoumacin A as it is produced and sequester it away from any *N*-acetylase enzymes produced by the bacteria. We subjected the crude extract to reversed-phase flash chromatography followed by reversed-phase high-performance liquid chromatography (RP-HPLC) to give amicoumacin A, along with amicoumacins B and C (Fig. 5). The structure of amicoumacin A was confirmed by LC-MS and comparison of the ^1^H NMR spectrum with that reported in the literature (43) (Fig. S8 to S10; Table S4). Unfortunately, we observed rapid, nonenzymatic conversion of the purified amicoumacin A to a mixture of amicoumacins, composed primarily of amicoumacin C, in addition to amicoumacin B and other derivatives (Table S5; Fig. S11 to S15). This transformation could be observed after just a couple of hours in a prepared LC-MS sample and seemed to be accelerated by trifluoroacetic acid that had been added to the HPLC eluent in order to get baseline separation of the amicoumacins. Thus, due to the limited amount of isolated amicoumacin A, we decided to perform downstream bioassay analysis with commercially available synthetic amicoumacins A and B, included as standards in the LC-MS analysis and molecular networking. MS^2^ spectra of in-house purified amicoumacin A and synthetic amicoumacins A and B can be found in Supplementary materials (Fig. S9, S16, and S17, respectively).

### MIC of amicoumacins A and B against *L. crescens*

**Fig 6:**
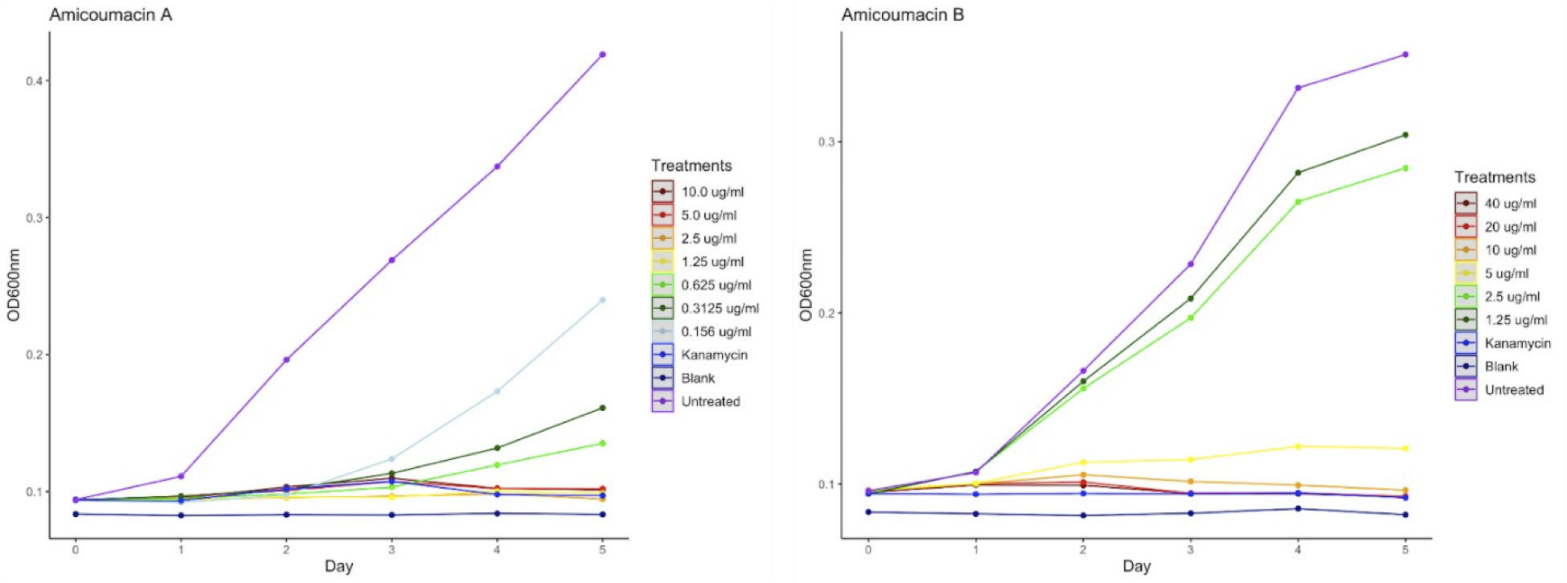
MIC of amicoumacin A (left) and amicoumacin B (right) to *L. crescens* (Lc). Growth of Lc was evaluated every 24 hours for 5 consecutive days. Points represent the average of six technical replicates per treatment, and the entire experiment was repeated twice. Determined MIC of amicoumacin A was 1.25 µg/mL (yellow line) and of amicoumacin B was 10 µg/mL (orange line).

Amicoumacin A has been reported to be highly effective against several gram-positive and gram-negative bacterial strains, including methicillin-resistant *Staphylococcus aureus* (MRSA) (45, 46), various *Bacillus* strains, and others (47, 48). We examined the effect of commercially available amicoumacin A and amicoumacin B against *L. crescens*, the surrogate bacterium for the HLB-associated pathogen, to determine the lowest dose at which no bacterial growth is observed, known as the MIC. Amicoumacin A showed strong inhibition to *L. crescens* growth *in vitro* over 5 consecutive days, with a MIC of 1.25 µg/mL (Fig. 6). This concentration was similar to the previously reported MIC (1.25 µg/mL) for the gram-negative shrimp pathogens belonging to *Vibrio* spp. (49). Furthermore, amicoumacin B was less inhibitory to *L. crescens* than amicoumacin A. The MIC value for amicoumacin B was 10 µg/mL, although there was moderate inhibition at 5 µg/mL. Each experiment included six technical replicates, and the experiment was repeated twice.

### Amicoumacin B and an isolated amicoumacin mixture are inhibitory to *C*Las

**Fig 7:**
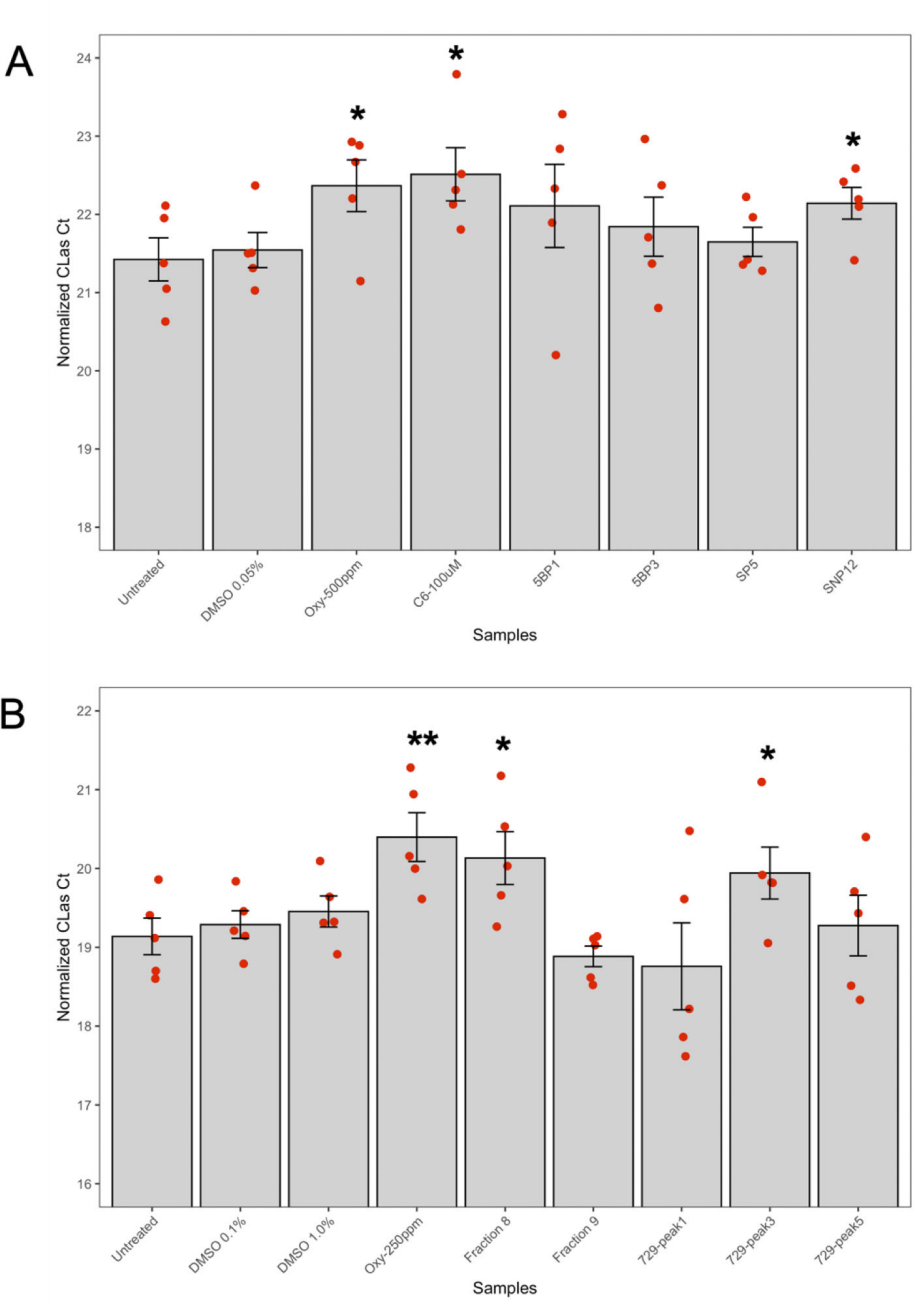
Amicoumacin-enriched fractions inhibited *C*Las in a citrus hairy root assay. Untreated and dimethyl sulfoxide (DMSO) (used to dissolve compounds) were used as negative control. OTC-treated hairy roots (250 and 500 ppm) and C6 were used as positive controls. (**A**) First assay of amicoumacin-enriched fractions indicated that fraction SNP12 (arbitrarily named) significantly reduced *C*Las titer. (**B**) Second assay of amicoumacin-enriched fractions indicated that fractions 8 and 729-peak1 (arbitrarily named) significantly reduced *C*Las titer. The bacterial titers were estimated by quantitative PCR (qPCR) after 72 h of treatment with each sample and plotted relative to those of untreated samples (set to 100%). Error bars represent the SEM (*n* = 5/treatment). *P*-values were calculated by a two-sample *t* test (one-tailed) relative to untreated samples. Samples that significantly reduced *C*Las titer compared to untreated are marked with an asterisk and represent *P*-value ≤ 0.05 (*) or *P*-value ≤ 0.01 (**). Each experiment contained five bioreplicates/fractions, and the entire experiment was repeated twice.

**Fig 8:**
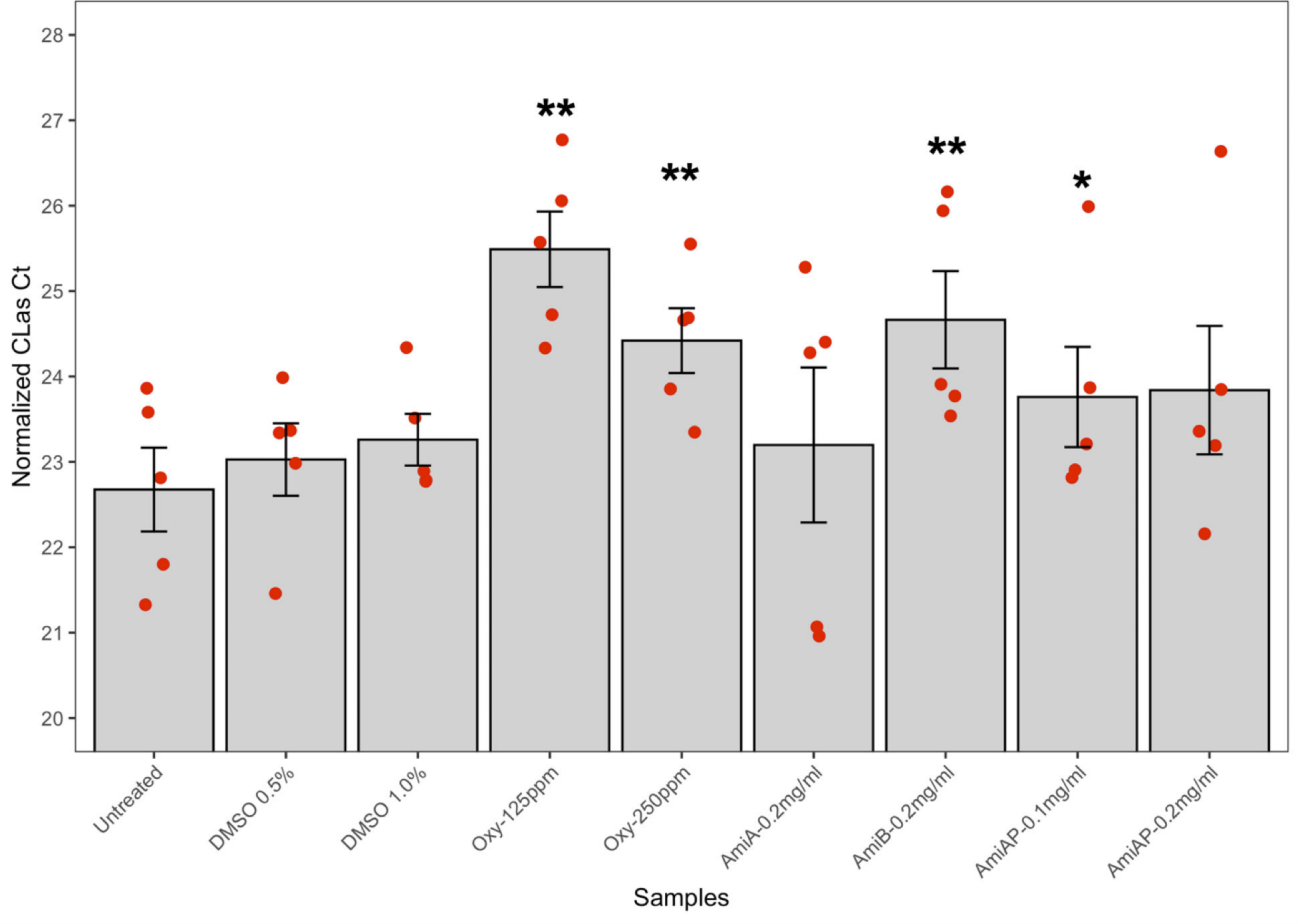
An amicoumacin mixture isolated from *B. safensis* and a commercial sample of amicoumacin B inhibit *C*Las in a citrus hairy root assay. Untreated (UT) and DMSO (0.5% and 1.0%), used to dissolve compounds, were used as negative control, and OTC-treated hairy roots (125 and 250 ppm) were used as positive control. Synthetic amicoumacin A (AmiA) and amicoumacin B (AmiB) were tested at 0.2 mg/mL, in addition to semi-purified amicoumacin A (AmiAP) from *B. safensis* CB729 at 0.1 and 0.2 mg/mL. The bacterial titers were estimated by quantitative PCR (qPCR) after 72 h of treatment with each sample and plotted relative to those of untreated samples (set to 100%). Error bars represent ±SEM (*n* = 5 bioreplicates/treatment). *P*-values were calculated by a two-sample *t* test (one-tailed) relative to untreated samples. Samples that significantly reduced *C*Las titer compared to untreated were marked with an asterisk and represented *P*-value ≤ 0.05 (*) or *P*-value ≤ 0.01 (**).

Amicoumacin-enriched fractions were screened against *C*Las in the hairy root assay. In two separate assays, three amicoumacin-enriched fractions (each with at least three technical replicates) significantly inhibited *C*Las, demonstrating that the suppressive effect of amicoumacins to *C*Las was repeatable (Fig. 7). To validate the effectiveness of amicoumacins A and B to *C*Las, we conducted a citrus anti-*C*Las hairy root assay, using *C*Las-infected citrus roots (50). We included the synthetic amicoumacins A and B and the amicoumacin mixture from *B. safensis* CB729 at different doses. We observed a significant reduction in *C*Las titer with amicoumacin B at 0.2 mg/mL (*P* ≤ 0.01) but not with amicoumacin A at the same concentration. We also observed a significant reduction in *C*Las titer from AmiAP (amicoumacin mixture) at 0.1 mg/mL (*P* ≤ 0.05; each treatment had five bioreplicates [Fig. 8]). Together, these results indicate the inhibitory effect of amicoumacins towards *C*Las in citrus tissue.

## DISCUSSION

Amicoumacins are a small group of natural products known for their broad antimicrobial properties against clinically relevant human pathogens, such as *Helicobacter pylori* (51) and MRSA. Amicoumacins belong to the dihydroisocoumarin group and are considered dipeptides, constructed of two amino acids (37). Their mode of action is to inhibit protein synthesis by stabilizing the interaction between the 16S rRNA and mRNA (52). Amicoumacins, including amicoumacin B, can also disrupt the quorum-sensing system of *Chromobacterium violaceum* ATCC12472 (53). In addition, they also have antiulcer and anticancer activities (54, 55). *Bacillus* is the predominant genus of bacteria to produce amicoumacins (37), but members of other genera, such as *Nocardia*, *Xenorhabdus*, and *Streptomyces*, can produce amicoumacins and their derivatives (56–58). Amicoumacin A is recognized as the most bioactive member of this group (43, 49). Meanwhile, amicoumacins B and C are mostly described as inactive compounds (41, 44, 45) but have been reported to display moderate antimicrobial activity (59). To our knowledge, there are only a few reports of the evaluation of amicoumacin-related compounds for their efficacy against plant pathogens. Specifically, *B. subtilis* cell-free supernatant, possibly containing lipopeptides and amicoumacin A, inhibited the growth of a citrus fruit fungus (60). In addition, xenocoumacin, an amicoumacin-related compound, was effective against *Phytophthora infestans* in potato plants (61).

Amicoumacin A-enriched fractions had significant inhibitory activity against *L. crescens* and *C*Las, so we initially sought to generate sufficient quantities of isolated amicoumacin A by large-scale fermentation of *B. safensis* CB729 to test against *L. crescens* and in downstream *C*Las inhibition assays using the citrus hairy root assay (50). Using two rounds of RP-HPLC, we successfully isolated amicoumacins A, B, and C from spent *B. safensis* CB729 broth. However, during this process, we observed the spontaneous cyclization of amicoumacin A into amicoumacin C and hydrolysis to amicoumacin B and other derivatives, which hindered our ability to test purified amicoumacin A isolated directly from the *B. safensis* CB729 strain in the downstream bioassays. The low stability of amicoumacin A and its conversion into less active derivatives, such as amicoumacins B and C, has been reported in the bacterial systems *S. aureus* and *B. subtilis* (42–44, 49, 54). Specifically, amicoumacins B and C were inactive against *S. aureus*, *B. subtilis*, and *Vibrio* spp., while amicoumacin A was highly active against these and other bacteria (41, 45, 49). While amides are usually quite recalcitrant to hydrolysis in physiological conditions, one hydroxy group in the structure of amicoumacin A is poised for an intramolecular esterification reaction to form the γ-lactone amicoumacin C, which in turn is susceptible to hydrolysis to amicoumacin B. Other examples in which an intramolecular mechanism accounts for the cleavage of amides even in mild conditions include the hydrolysis of *N*-acylated peptide derivatives (62) and the classic Edman degradation method for sequencing peptides (63). This non-enzymatic transformation appeared to proceed more rapidly in the presence of trifluoroacetic acid, which was added to the HPLC eluent to achieve baseline separation of the amicoumacins.

Because of the rapid conversion of amicoumacin A isolated from *B. safensis* CB729, we tested commercially available synthetic amicoumacins A and B against *L. crescens* to obtain the MICs for these compounds against *L. crescens*. Amicoumacin A was more effective against *L. crescens* in the *in vitro* assay, with an MIC of 1.25 µg/mL, whereas the MIC value for amicoumacin B was 10 µg/mL. Interestingly, in the *ex vivo* hairy root assay, we observed essentially the opposite, where synthetic amicoumacin B was significantly inhibitory to *C*Las, and synthetic amicoumacin A was not significantly inhibitory. Amicoumacin A has a terminal amide group, whereas amicoumacin B has the corresponding carboxylic acid, which renders it more water soluble. Because of this, we speculate that amicoumacin B may be able to penetrate the plant tissue more readily than amicoumacin A, making it more effective at accessing *C*Las in the hairy root assay. Interestingly, the amicoumacin mixture that contained amicoumacins A and B, as well as amicoumacin C and other derivatives isolated from *B. safensis* CB729, was inhibitory to *C*Las *ex vivo. N*-acetylamicoumacin C was not active against *L. crescens* (data not shown). These data, taken together, suggest that amicoumacin B is more bioactive against *C*Las than amicoumacin A in the citrus hairy root assay.

Culture collections are an important translational research tool to bridge DNA-based microbiome data sets with biologically relevant activities (29, 64). Curated microbial resources enable critical inquiries into specific microbial interactions, such as linking functional phenotypes like pathogen suppression to specific microbiome constituents and their respective bioactive chemistries. *B. safensis* has been identified as a plant growth-promoting bacterium and a biocontrol agent in other systems (65–68). Here, we describe the anti-*Liberibacter* activity of *B. safensis* CB729 mined from the citrus microbiome. The next steps of this collective work are to evaluate *B. safensis* CB729 live inoculum and amicoumacins for anti-*C*Las activity within citrus trees. Our overall goal is to determine how the citrus microbiome interfaces with the *C*Las pathogen and eventually to understand the impact of microbial community composition on HLB outcomes. In the long term, these findings will lay the foundation for the development of sustainable plant disease mitigation strategies for Huanglongbing and commercial citriculture that can be used in rotation with or in place of OTC applications.

## MATERIALS AND METHODS

### Bacterial isolates and growth conditions

*B. safensis* CB729 was isolated from the leaf tissue of citrus trees in Florida, USA, under high HLB disease pressure. A sampling of plant material, bacterial isolation, and genus-level identification was initiated as a component of a bioprospecting effort designed to generate a citrus-associated microbial repository and is described in detail by (29). *B. safensis* CB729 was recovered from glycerol stocks and cultivated in tryptic soy agar at 28°C for 3 days and propagated in liquid culture of tryptic soy broth (TSB), incubated at 28°C at 180 rpm.

### Whole-genome sequencing, assembly, and annotation

The whole-genome sequencing, assembly, and annotation of *B. safensis* CB729 are described in reference 39. Briefly, DNA was extracted from a single colony grown overnight at 28°C in TSB, using Wizard Genomic DNA Purification Kit (Promega Corporation, Madison, Wisconsin, USA), following the protocol for gram-positive bacteria. Library preparation and sequencing were performed by SeqCenter (Pittsburgh, Pennsylvania, USA). Sequence analysis from quality control to annotation was performed on the KBase web service (69), and the publicly available narrative containing analyses and data can be found at https://doi.org/10.25982/157793.280/2368552. Default parameters were used, except where noted. Quality of raw reads was assessed using FastQC (v0.12.1), and quality control was performed using JGI RQCFilter pipeline BBTools (v38.22) and PRINSEQ (v0.20.4). Genome assembly was performed on SPAdes (v3.15.3) with k-mer sizes set for 21, 33, 55, 77, 91, and 111, and the quality of assembly was assessed with QUAST (v4.4) and CheckM (v1.0.18). The genome was annotated using Prokka (v1.14.5), and taxonomic identification was performed on GTDB-Tk (v2.3.2). The phylogenetic tree was generated with Species Tree (v2.2.0) and annotated on iTOL (v6.8.1) (70). Identification of biosynthetic gene clusters was performed with antiSMASH v7.0 (71). Gene cluster comparison analysis was performed on the clinker entry point of Cagecat (72).

### Isolation of secondary metabolites from *B. safensis* CB729

*B. safensis* CB729 crude extract was initially produced by inoculating a single colony of CB729 in A21 media (20 g/L D-glucose, 5 g/L yeast extract, 1 g/L K_2_HPO_4_, 0.5 g/L MgSO_4_:7 H_2_O, 0.5 g/L KCl, 1.6 mg/L CuSO_4_, 1.2 mg/L Fe_2_(SO_4_)_3_, and 0.4 mg/L MnSO_4_) (73). The culture was incubated at 28°C, 180 rpm for 72 h. The fermentation broth was centrifuged at 10,000 × *g* for 10 min, and the supernatant was extracted three times by liquid:liquid partitioning with two volumes of ethyl acetate. The organic layer was pooled and concentrated on a rotary evaporator (Rotavapor, R-200, BÜCHI, Flawil, Switzerland), yielding a crude extract of 164.2 mg. The crude extract was loaded into RediSep Rf Gold 12 g HP Silica Column (Teledyne, ISCO, Nebraska, USA) and subjected to normal phase high-performance liquid chromatography on a CombiFlash EZ-Prep (Teledyne, ISCO, Nebraska, USA) with a gradient elution (20%–100% hexane:ethyl acetate and 0%–20% dichloromethane:methanol) over 35 min, flow rate: 22 mL/min. All fractions were collected and tested against *L. crescens* in an established inhibition bioassay.

### High-Resolution mass spectrometry and NMR analysis

Fraction six from flash chromatography was subjected to reversed-phase HPLC (Prominence-i LC-2030C liquid chromatograph equipped with a diode-array detector; Shimadzu Scientific Instruments) on a Luna C18(2) semi-preparative column (5 µm × 10 mm × 250 mm) with gradient elution (45%–60% acetonitrile:water over 8 min; ramped to 100% over 5 min) to give pure *N*-acetylamicoumacin C (0.8 mg, *t*_*R*_ = 10.2 min). Liquid chromatography-electrospray ionization mass spectrometry was performed on an HPLC system (Agilent, Model 1260 Infinity) that was equipped with a degasser, binary pump, autosampler, and diode array detector, coupled to a QToF device (Agilent, Model 6530 Accurate-Mass QToF) with an electrospray ionization (ESI) source. 1D ^1^H NMR and 2D (^1^H-^1^H COSY, ^1^H-^13^C HSQC, and ^1^H-^13^C HMBC) NMR spectra were obtained using a JEOL ECS spectrometer (400 MHz for ^1^H and 100 MHz for ^13^C) using CDCl_3_ from Cambridge Isotope Laboratories, Inc., and referenced to tetramethylsilane.

### Molecular networking

A molecular network was created with the FBMN workflow (74) on GNPS2 (https://gnps2.org) (75). The mass spectrometry data were first converted to open format files (mzML) using DataConnect, available from the Waters MicroApps website. The files were then processed with MZMINE 3.9.0 (76), and the results were exported to GNPS2 for FBMN analysis. The data were filtered by removing all MS/MS fragment ions within ±17 Da of the precursor m/z. MS/MS spectra were window filtered by choosing only the top six fragment ions in the ±50 Da window throughout the spectrum. The precursor ion mass tolerance was set to 0.02 Da, and the MS/MS fragment ion tolerance was set to 0.02 Da. A molecular network was then created in which edges were filtered to have a cosine score above 0.7 and more than six matched peaks. Furthermore, edges between two nodes were kept in the network if and only if each of the nodes appeared in each other’s respective top 10 most similar nodes. Finally, the maximum size of a molecular family was set to 100, and the lowest-scoring edges were removed from molecular families until the molecular family size was below this threshold. The spectra in the network were then searched against Global Natural Products Social Molecular Networking (GNPS) spectral libraries (75, 77). The library spectra were filtered in the same manner as the input data. All matches kept between network spectra and library spectra were required to have a score above 0.7 and at least six matched peaks. The DEREPLICATOR was used to annotate MS/MS spectra (78). The molecular networks were visualized using Cytoscape software v. 3.9.1 (79).

### Large-scale production and purification of amicoumacins

Experimentation with different growth conditions revealed high amicoumacin yield from *B. safensis* CB729 growth in SYC medium (containing 40 g/L sucrose, 5 g/L yeast extract, 4 g/L CaCO_3_, 1.5 g/L K_2_HPO_4_, 2 g/L glucose, 2 g/L NaCl, 1.5 g/L MgSO_4_, 2 g/L [NH_4_]_2_SO_4_, 0.01 g/L FeSO_4_, and 0.01 g/L MnCl_2_) (44). A single colony was grown overnight in TSB at 28°C, and 1 mL of the overnight culture was used to inoculate each of 12 × 1 L of fresh SYC medium in 2.5 L Ultra Yield flasks (Thomson) containing Diaion HP-20 resin (20 g/L) resin and incubated at 28°C, 180 rpm for 48 h. The resin was collected using cheesecloth and rinsed with deionized water. The resin was transferred to a beaker, covered with acetone, and stirred for 1 hour, and the mixture was filtered using cheesecloth to give an acetone extract. The resin beads were extracted a second time with acetone, the extracts were combined, and the volume was reduced on a rotary evaporator (Rotavapor, R-210, BÜCHI, Flawil, Switzerland) to leave a concentrated residue. The residue (200 mL) was loaded onto a RediSep Rf C-18 SPE Cartridge (Teledyne ISCO, Nebraska, USA), rinsed with deionized water, and subjected to flash column chromatography on a CombiFlash Rf + LC system (Teledyne ISCO) using a RediSep Gold C18 Reversed Phase Column (30 g, Teledyne ISCO) with gradient elution (50% aqueous methanol up to 100% methanol, with isocratic pauses at each peak; total run time 32.6 min) at a flow rate of 35 mL/min. Fraction E (267 mg) eluted at 72% aqueous methanol and exhibited NMR signals consistent with the amicoumacins. This fraction was subjected to reversed-phase HPLC (Prominence-i LC-2030C liquid chromatograph equipped with a diode-array detector; Shimadzu Scientific Instruments) on a Luna C18(2) semi-preparative column (5 µm × 10 mm × 250 mm) with isocratic elution (20% aqueous acetonitrile containing 0.01% trifluoroacetic acid) to give amicoumacins A (34.0 mg, *t*_*R*_ = 7.4 min), B (6.2 mg, *t*_*R*_ = 10.0 min), and C (1.2 mg, *t*_*R*_ = 11.4 min).

### Mass spectrometry analysis

LC-MS/MS analysis of *B. safensis* CB729 crude extract and fractions was performed at the University of California Riverside Metabolomics Core Facility as described previously (80) with minor modifications. Briefly, analysis was performed on a Synapt G2-Si quadrupole time-of-flight mass spectrometer (Waters, Milford, MA, USA) coupled to an Acquity I-class UPLC system (Waters). Separations were carried out on a CSH phenyl-hexyl column (2.1 × 100 mm, 1.7 µM; Waters). The mobile phases were (i) water with 0.1% formic acid and (ii) acetonitrile with 0.1% formic acid. The flow rate was 250 µL/min, and the column was held at 40°C. The injection volume was 1 µL. The gradient was as follows: 0 min, 1% B; 1 min, 1% B; 8 min, 40% B; 24 min, 100% B; 26.5 min, 100% B; 27 min, 1% B; 30 min, 1% B. The MS scan range was (50–1,200 or 1,600/z) with a 100 ms scan time. MS/MS was acquired in a data-dependent fashion. Source and desolvation temperatures were 150°C and 600°C, respectively. Desolvation gas was set to 600 L/h, and cone gas was set to 0 L/h. All gases were nitrogen except the collision gas, which was argon. The capillary voltage was 1 kV in positive ion mode. Leucine enkephalin was infused and used for mass correction. Waters raw files (.raw) were converted to standard output format (mzML) via Waters microapp and DataConnect (https://microapps.on-demand.waters.com/).

### *L. crescens* inhibition bioassay

*B. safensis* CB729 crude extract and step-purification fractions were tested against *L. crescens* in an agar diffusion inhibition bioassay (29). Briefly, crude extract or fractions were resuspended in 100% MeOH and 15 µL applied to sterile paper discs (Becton, Dickinson, Franklin Lakes, NJ) and allowed to dry in a biosafety cabinet. *L. crescens* was cultivated in bBM7 plus 1.0 methyl-β-cyclodextrin (mβc; 1 g/L methyl-β-cyclodextrin, 2 g/L α-ketoglutarate, 10 g/L aces buffer, and 3.75 g/L potassium hydroxide) (81) liquid medium at 28°C, 150 rpm for up to 4 days. bBM7 plus mβc top agar (0.8% agar) was prepared, cooled to 60°C, and amended with a 4-day *L*. *crescens* liquid culture at 10% of the top agar volume.

### MIC of amicoumacins A and B against *L. crescens*

The MIC was calculated for amicoumacin A and B in liquid bBM7 plus mβc as described previously in reference 82. In brief, amicoumacins A and B were individually dissolved in methanol and added to a 96-well plate. Methanol was evaporated in a biosafety cabinet overnight. The following day, 150 µL of sterile bBM7 plus mβc was added to the wells, along with 50 µL of 4-day-old liquid culture (OD600_nm_ ∼ 0.27) of *L. crescens*. The plate was incubated at 28°C at 150 rpm. Absorbance was read at OD600_nm_ using an Infinite 200 Pro plate reader (Tecan Group Ltd., Switzerland) daily for 5 days. The lowest dose at which no growth was observed was recorded as the MIC. Each treatment had six technical replicates, and the experiment was repeated two times for each compound.

### Citrus anti-CLas *C*Las hairy root assay

The anti-*C*Las hairy root assay was performed using HLB-confirmed sour orange citrus tissues (*Citrus x aurantium* L.). Briefly, citrus budwood tissues were transformed with *Rhizobium rhizogenes* to induce hairy roots (50). The presence of *C*Las in the emerging hairy roots was verified by quantitative PCR (qPCR) using primers specific to the *C*Las ribonucleotide reductase β-subunit (*nrdB*) gene (83). Next, *C*Las-citrus hairy roots were collected, surface-sterilized with 70% ethanol and 1% bleach, and transferred into multi-well plates with Gamborg’s B-5 medium containing 1% sucrose. Synthetic amicoumacin A (AmiA) and amicoumacin B (AmiB) were tested at 0.2 mg/mL, in addition to amicoumacin mixture (AmiAP) from *B. safensis* CB729 at 0.1 and 0.2 mg/mL. Treatments were vacuum infiltrated and incubated at 25°C in the dark for 72 hours, and each treatment included five biological replicates. OTC hydrochloride treatment was used as a positive control alongside untreated or mock (DMSO) negative controls. After 72 hours, the hairy root tissue was treated with propidium monoazide (PMAxx, Biotium) dye to inactivate DNA from dead *C*Las. Total DNA was extracted, and viable bacterial titers were measured by qPCR using primers specific to the *C*Las (nrdB) (83). Raw *C*Las Ct values were further normalized to an endogenous citrus housekeeping gene, the glyceraldehyde-3-phosphate dehydrogenase 2 (*GAPC2*) gene (50, 84), and the relative levels were compared to untreated control.

## Data Availability

The data that support the findings of this study are openly available in the GenBank BioProject under no. PRJNA1046128, SRA accession number SRR26973669, and WGS accession number JAXKIG000000000. The link for the feature-based molecular networking (FBMN) generated on GNPS2 is available here: GNPS2 network link.

## SUPPLEMENTAL MATERIAL

Supplemental material aem.00869-25-s0001.pdf Tables S1 to S5; Figures S1 to S17.

ASM does not own the copyrights to Supplemental Material that may be linked to, or accessed through, an article. The authors have granted ASM a non-exclusive, world-wide license to publish the Supplemental Material files. Please contact the corresponding author directly for reuse.
