# Supplementary material for: Amicoumacins produced by the native citrus microbiome isolate Bacillus safensis inhibit the Huanglongbing-associated bacterial pathogen “Candidatus Liberibacter asiaticus”
Source: Appl Environ Microbiol. 2025 Jul 31;91(8):e00869-25. doi: 10.1128/aem.00869-25 (PMC12366304; doi:10.1128/aem.00869-25)

Supplemental Material

Amicoumacins produced by the native citrus microbiome isolate, *Bacillus safensis*, inhibit the Huanglongbing-associated bacterial pathogen, '*Candidatus* Liberibacter asiaticus'

Flavia Campos Vieira[1], Kranthi K. Mandadi[2,3,4,] Manikandan Ramasamy[2], Amancio de Souza[5], Kiana Callahan[6], Corrie Fyle[6], Andrew Kamemoto[6], Amanda G. Koontz[6], Christopher Yang[6], Robert Crowley III[7], Kevin G. M. Kou[7], Katherine N. Maloney[6], and M. Caroline Roper[1#]

[1]Department of Microbiology and Plant Pathology, University of California, Riverside, CA, USA.
[2]Texas A&M AgriLife Research & Extension Center, Texas A&M University System, Weslaco, TX, USA.
[3]Department of Plant Pathology and Microbiology, Texas A&M University, College Station, TX, USA.
[4]Institute for Advancing Health Through Agriculture, Texas A&M AgriLife, College Station, TX, USA
[5]Metabolomics Core Facility, Institute for Integrative Genome Biology, University of California Riverside, CA, USA.
[6]Department of Chemistry, Point Loma Nazarene University, San Diego, CA, USA.
[7]Department of Chemistry, University of California, Riverside, CA, USA.

List of contents

Figure S1) *In vitro* antagonistic activity of 10 citrus bacterial isolates against *Liberibacter crescens* in agar diffusion bioassay.

Figure S2) Circular representation of *Bacillus safensis* CB729 genome for specific genome features.

Table S1) Genome assembly statistics of *B. safensis* CB729.

Table S2) ^{1}H and ^{13}C NMR chemical shift comparisons for isolated **N-acetylamicoumacin C** with literature values.

Table S3) *Bacillus safensis* CB729 crude extract production and anti-*L. crescens* bioassay-guided HPLC fractionation.

Figure S3) LC-MS data for isolated **N-acetylamicoumacin C**.

Figure S4) ^{1}H NMR spectrum (400 MHz, $CDCl_3$) of isolated **N-acetylamicoumacin C**.

Figure S5) ^{1}H-^{13}C HSQC spectrum ($CDCl_3$) of isolated **N-acetylamicoumacin C**.

Figure S6) ^{1}H-^{13}C HMBC spectrum ($CDCl_3$) of isolated **N-acetylamicoumacin C**.

Figure S7) Entire featured-based molecular networking (FBMN) from LC-MS/MS data of the crude extract of *Bacillus safensis* CB729 generated using GNPS2.

Table S4) ^{1}H NMR chemical shift comparison for isolated **amicoumacin A** with literature values.

Figure S8) LC-MS Chromatograms: TIC and UV_{254} for isolated **amicoumacin A**.

Figure S9) Mass spectrum (MS) for isolated **amicoumacin A**.

Figure S10) ^{1}H NMR spectrum (400 MHz, CD_3OD) for isolated **amicoumacin A**.

Table S5) ^{1}H and ^{13}C NMR chemical shifts of **amicoumacin C** as the major decomposition product of sample of purified amicoumacin A.

Figure S11) LC-MS data of the decomposed sample of amicoumacin A, showing **amicoumacin C** as the major component.

Figure S12) ^{1}H NMR data of **amicoumacin C** as the major decomposition product of a sample of purified amicoumacin A.

Figure S13) ^{13}C NMR spectrum (100 MHz, DMSO- d_6) for isolated **amicoumacin C** as the major decomposition product of a sample of purified amicoumacin A.

Figure S14) ^{1}H-^{13}C HMBC spectrum (400 MHz, DMSO- d_6) for isolated **amicoumacin C** as the major decomposition product of a sample of purified amicoumacin A.

Figure S15) ^{1}H-^{13}C HMBC spectrum (400 MHz, DMSO-d_6) for isolated **amicoumacin C** as the major decomposition product of a sample of purified amicoumacin A, zoomed in to show the correlation from H-9’ to C-12’.

Figure S16) (A) Extracted ion chromatogram of synthetic **amicoumacin A**. (B) MS2 compound analysis of synthetic **amicoumacin A**.

Figure S17) (A) Extracted ion chromatogram of synthetic **amicoumacin B**. (B) MS2 compound analysis of synthetic **amicoumacin B**.

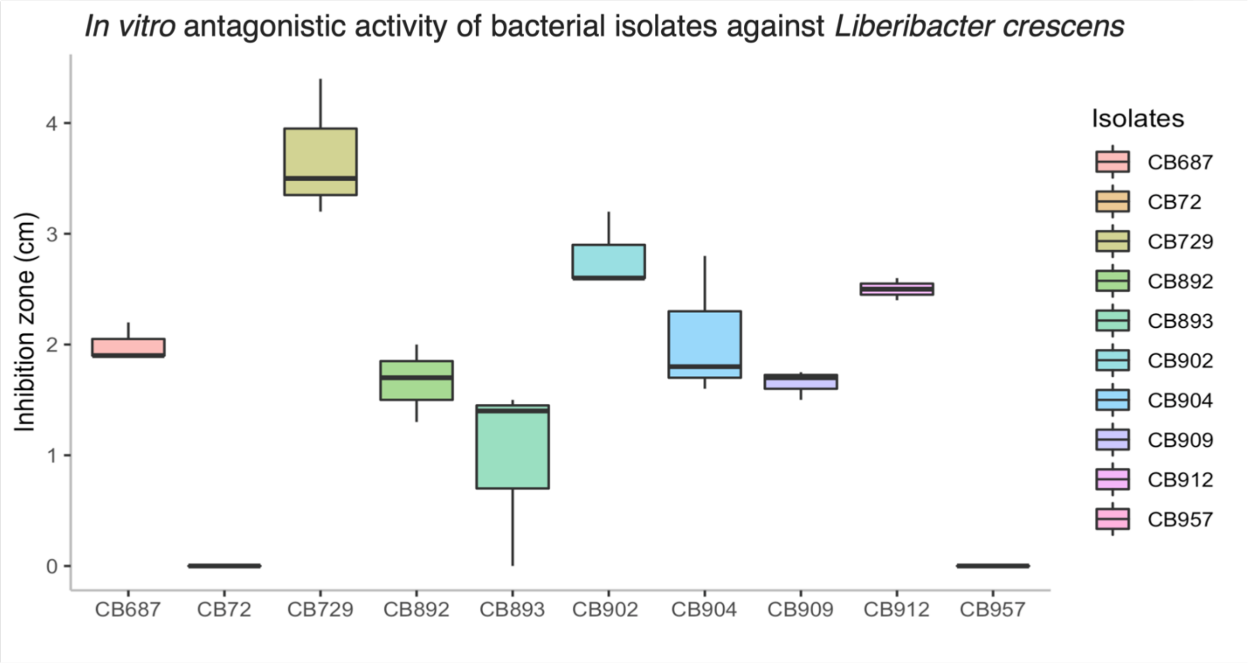

Figure S1 - *In vitro* antagonistic activity of 10 bacterial isolates from the citrus microbiome against *L. crescens* in agar diffusion bioassay. Boxplots show an average of three replicates.

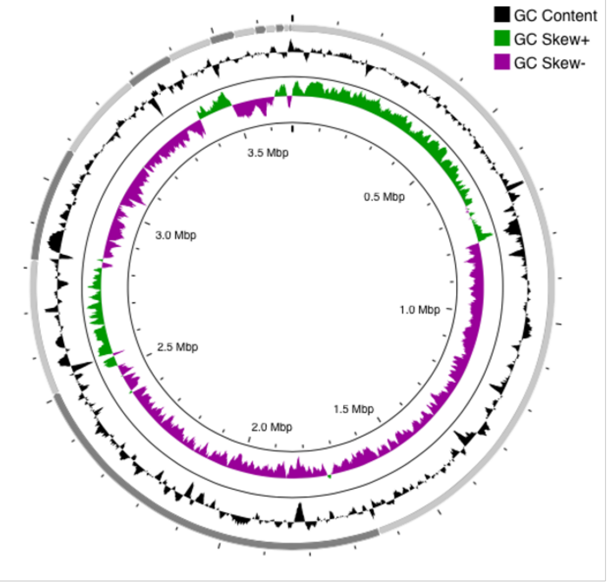

Figure S2 - Circular representation of *B. safensis* genome CB729 for specific genome features. Circles display the following, from the inside out: (1) Negative GC skew (pink), (2) positive GC skew (green) and (3) GC content (black). Map generated with Proksee software.

Table S1 – Genome assembly statistics of *B. safensis* CB729 (GenBank accession no: GCA_036621555.1).

**Features**	**Value**
Genome topology	Circular
Genome Size (bp)	3,677,118
N50 (bp)	871,191
Number of contigs	22
GC content (%)	41.50
Number of predicted genes	3,763
Protein coding sequences (CDS)	3,654

Table S2 - ^{1}H and ^{13}C NMR chemical shift comparisons for isolated ***N*-acetylamicoumacin C** with literature values.

	Measured in $CDCl_3$		Reported in Park *et al.* 2016, CD_3OD	
position	δ_C, type[a]	δ_H, mult, (J in Hz)	δ_C	δ_H, mult, (J in Hz)
1	[b]	-	169.5	-
3	80.9, CH	4.65, *dt* (12.0, 3.2)	81.1	4.67, *dt* (11.3, 3.8)
4	30.7, CH_2	3.02, *dd* (16.5, 12.0);<br>2.90, *dd* (16.5, 3.2)	29.4	2.95, *m*
5	118.4, CH	6.71, *d* (7.6)	118.0	6.79, *d* (8.0)
6	136.8, CH	7.43, *dd* (8.4, 7.6)	136.0	7.45, *t* (8.0)
7	116.6, CH	6.89, *d* (8.4)	115.2	6.83, *d* (8.0)

8	162.2, C	-	161.7	-
8-OH	-	10.75	-	-
9	108.0, C	-	107.9	-
10	139.0, C	-	139.8	-
1’	23.3	0.97, *d* (6.6)	22.3	0.95, *d* (6.7)
2’	21.8	0.91, *d* (6.4)	20.3	0.88 *d* (6.5)
3’	24.6	1.63[c]	24.2	1.62, *m*
4’	40.3	1.80, *ddd* (13.9, 10.3, 4.9)<br>1.44, *ddd* (13.9, 9.3, 4.8)	39.0	1.82, *m*;<br>1.41, *ddd* (13.9, 9.8, 4.2)
5’	49.8	4.30, *m*	49.0	4.30, *dt* (10.9, 3.9)
7’	[b]	-	171.4	-
8’	71.4	4.58, *d* (2.4)	71.8	4.41, *d* (2.4)
9’	85.0	4.41, *m*	85.8	4.76, *t* (2.4)
10’	45.9	4.35	46.6	4.49, *dt* (8.9, 2.5)
11’	36.3	3.11, *dd* (18.2, 9.0);<br>2.51, *dd* (18.2, 5.4)	35.9	3.03, *dd* (18.3, 8.9);<br>2.35, *dd* (18.3, 2.7)
12’	174.8	-	176.4	-
14’	170.7	-	172.4	-
15’	23.1	1.93	20.8	1.85, *s*

[a] Measured indirectly from HSQC & HMBC spectra
[b] Not observed
[c] Overlapped with water signal at 1.56

Table S3 - The table indicates 7 independent HPLC fractionation runs of crude extracts produced by *B. safensis* CB729. In all runs, the anti-*L. crescens* fractions were enriched in amicoumacins. For each of the 7 bioreplicates, there were 3 technical replicates.

Run	Media	Growth conditions	Sample loaded (mg)	HPLC mode	Bioactive fractions	Bioactive fractions with amicoumacins
1	A21	1L, 28°C, 180 rpm, 3 days	164.9	normal	7	2
2	A21	1L, 28°C, 180 rpm, 3 days	3,120	normal	6	4

3	A21/resin	1L, 28°C, 180 rpm, 7 days	298.4	normal	5	4
4	SYC	1L, 28°C, 180 rpm, 2 days	-	normal	6	3
5	A21	500 ml, 28°C, 180 rpm, 3 days	135.0	reverse	2	2
6	A21	500 ml, 28°C, 180 rpm, 3 days	176.9	reverse	3	3
7	A21	500 ml, 28°C, 180 rpm, 3 days	50.0	reverse	8	5

Figure S3) LCMS data for isolated ***N*-acetylamicoumacin C**: UV_{254} (top), Total Ion Chromatogram (TIC, middle), and mass spectrum (bottom):

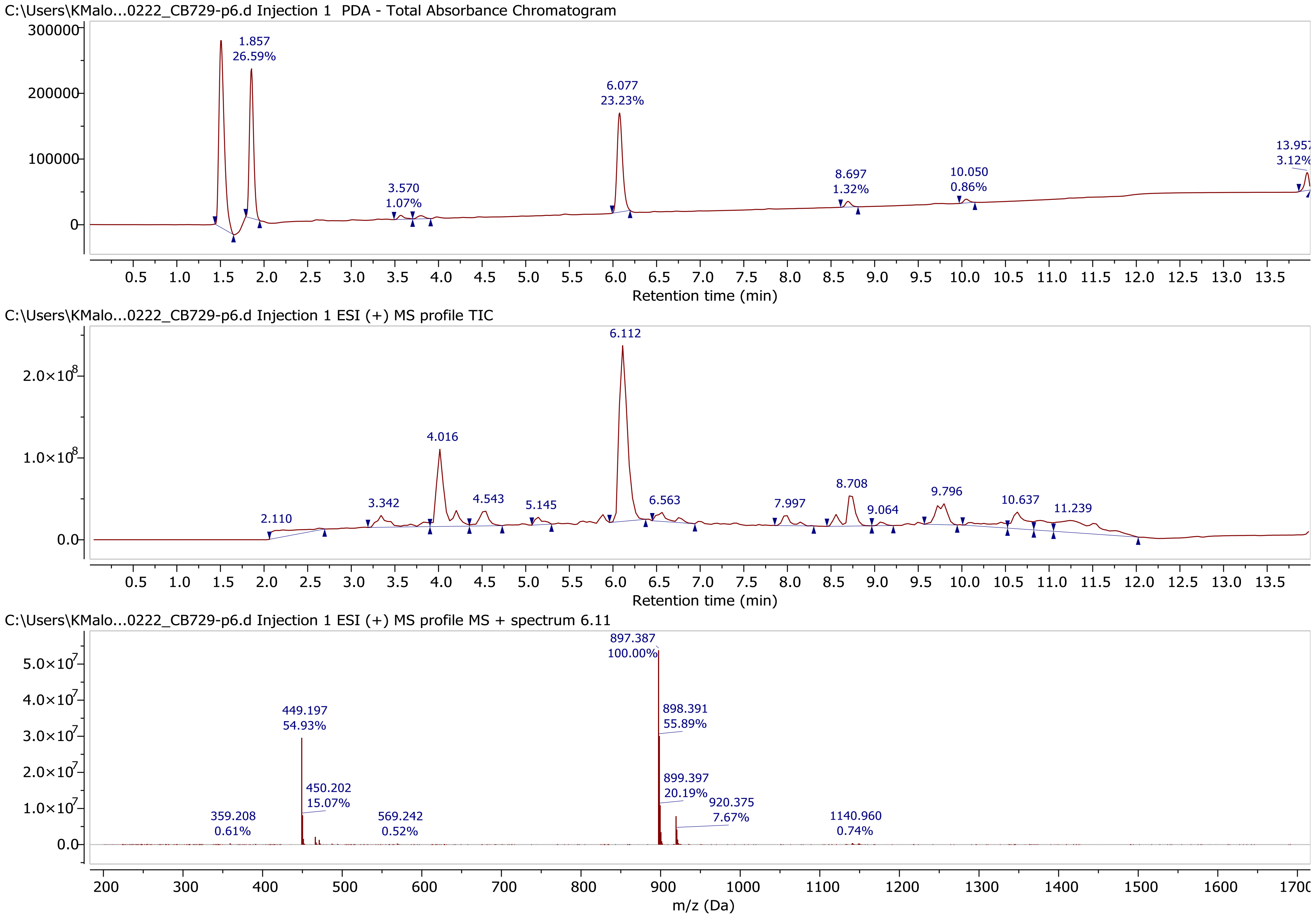

Figure S4) ^{1}H NMR spectrum (400 MHz, $CDCl_3$) of isolated ***N*-acetylamicoumacin C.**

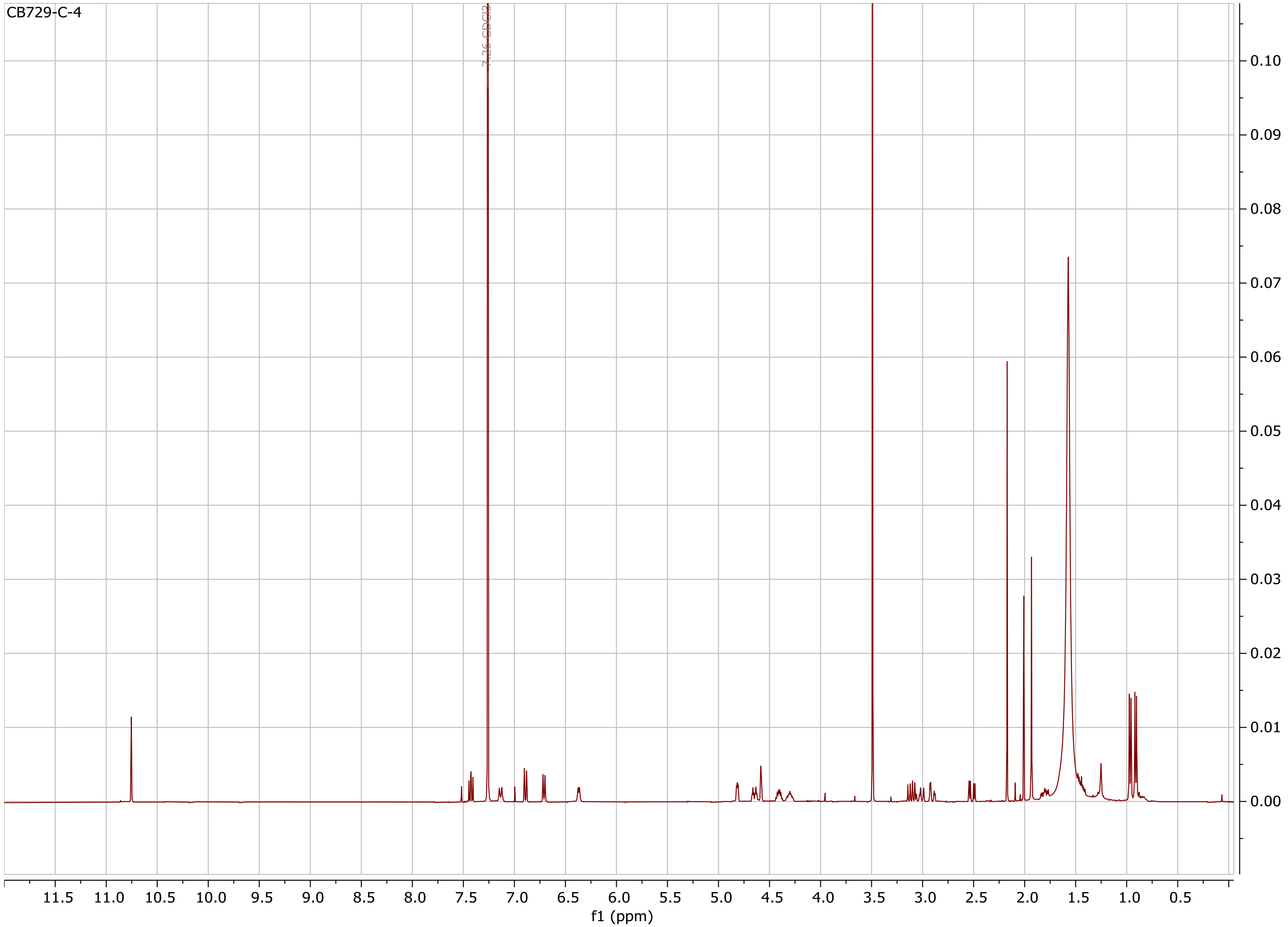

Figure S5) ^{1}H-^{13}C HSQC spectrum ($CDCl_3$) of isolated ***N*-acetylamicoumacin C.**

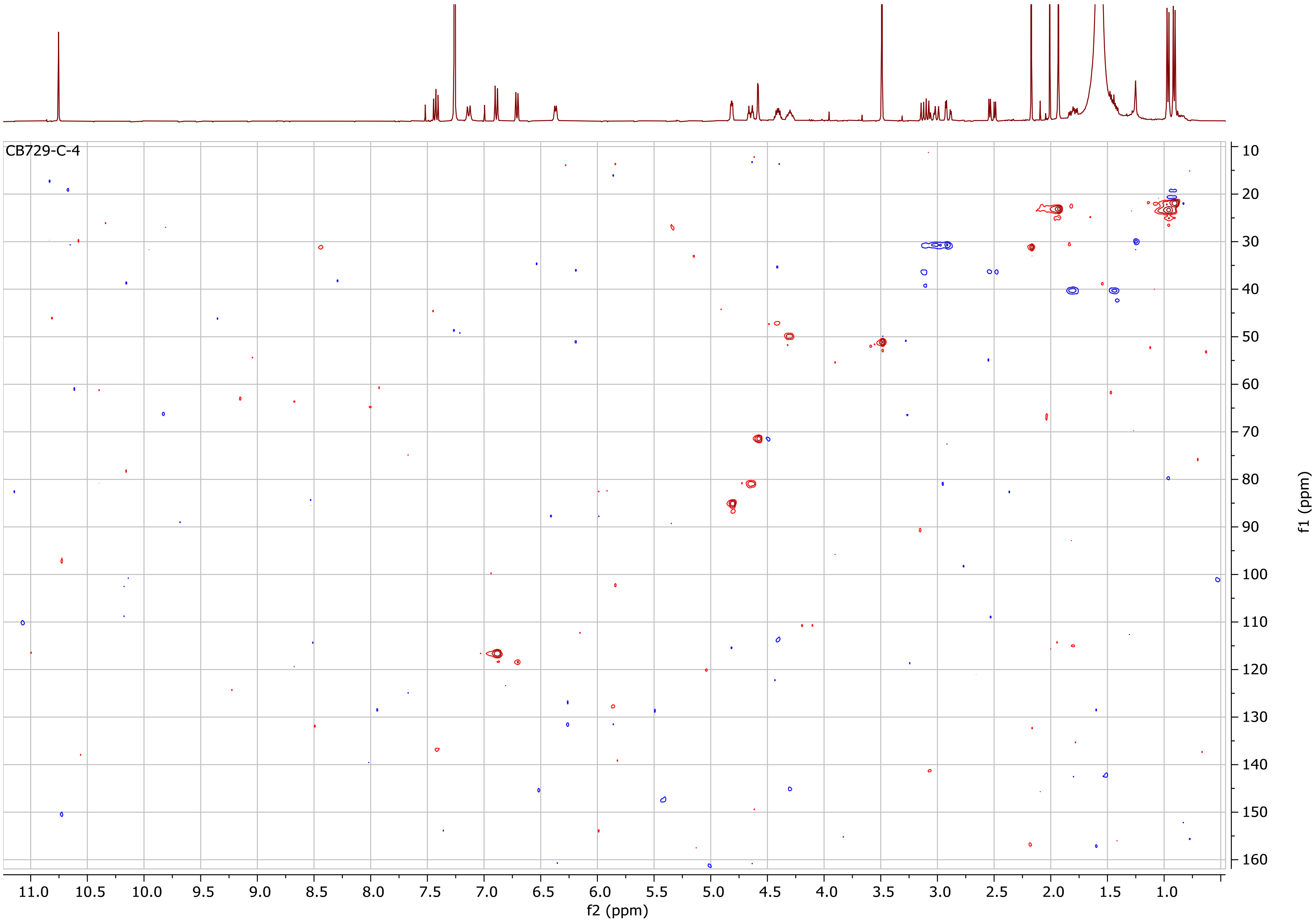

Figure S6) ^{1}H-^{13}C HMBC spectrum ($CDCl_3$) of isolated ***N*-acetylamicoumacin C.**

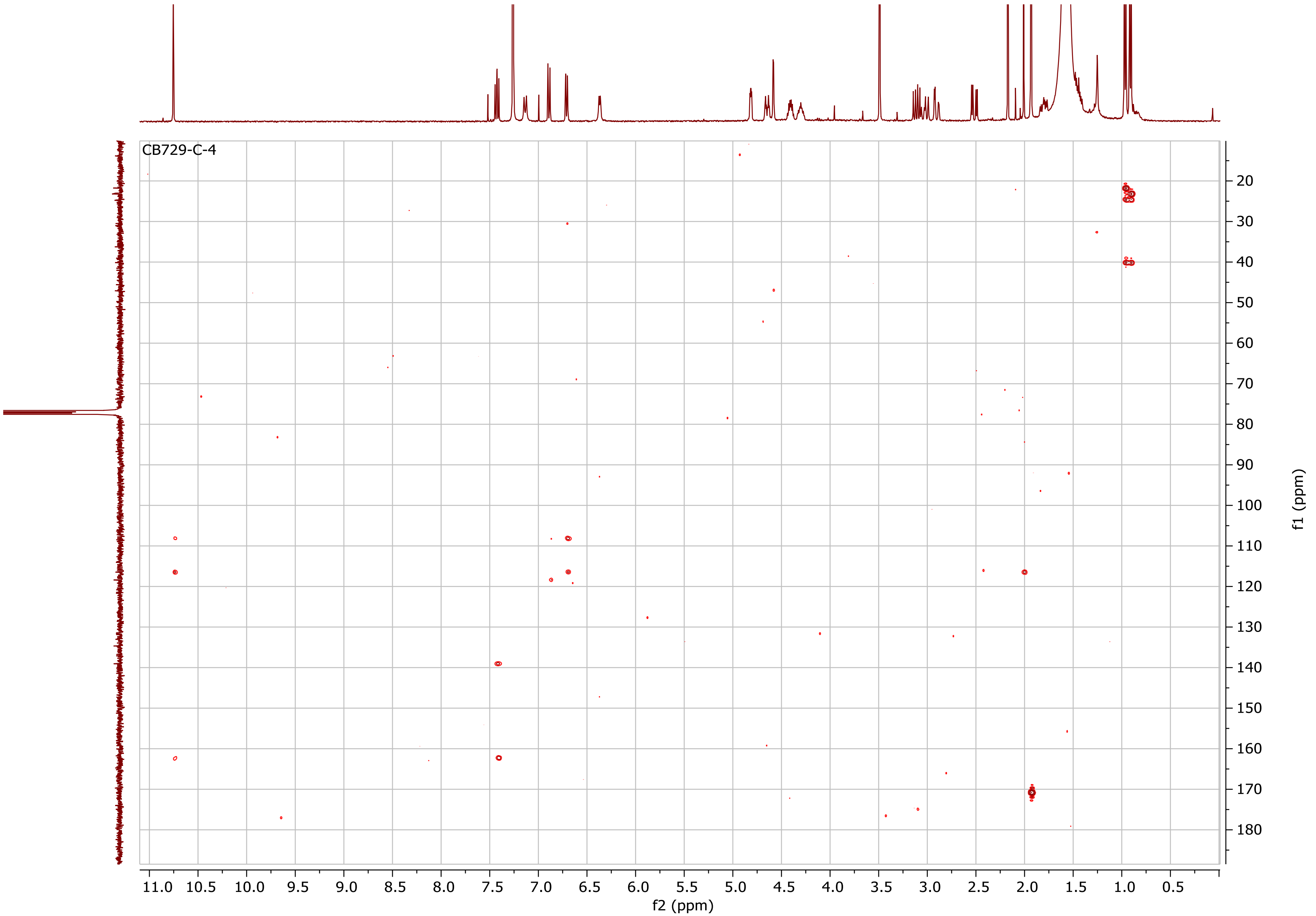

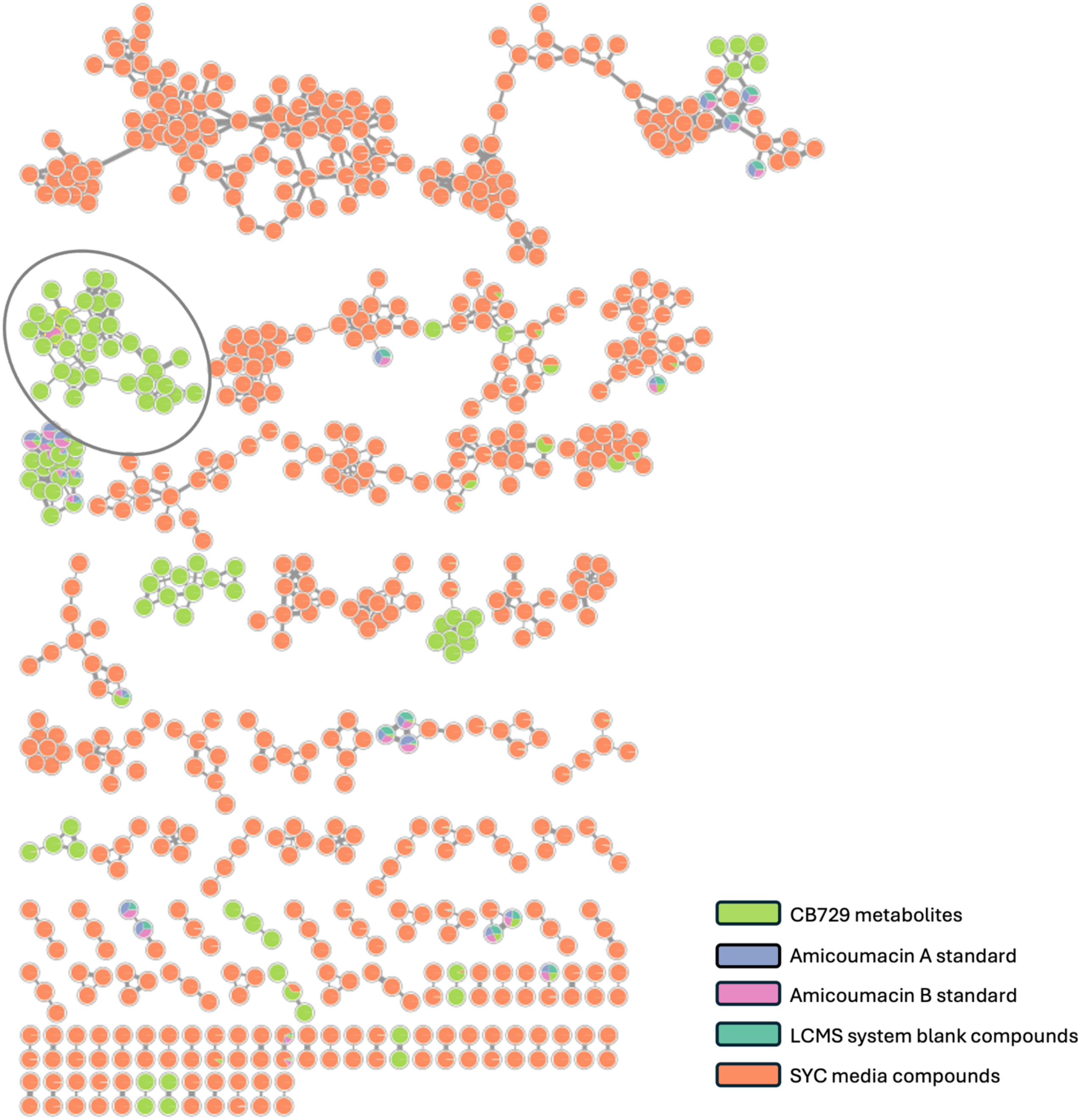

Figure S7 - Entire featured-based molecular networking (FBMN) from LC-MS/MS data of the crude extract of *Bacillus safensis* CB729 generated using GNPS2. Nodes are colored according to their origin, described in the legend box. Molecular family containing amicoumacins are highlighted in a circle.

Table S4) ^{1}H NMR chemical shift comparison for isolated **amicoumacin A** with literature values.

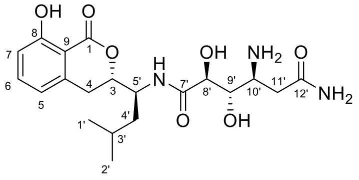

	Measured in CD_3OD	Reported in Park *et al*. 2016, DMSO-d_6
position	δ_H, mult, (J in Hz)	δ_H, mult, (J in Hz)
3	4.68, *dt* (11.5, 3.6)	4.69, *m*
4	3.06, *dd* (16.4, 12.0);<br>2.97, *dd* (16.4, 3.5)	3.00, *dd* (16.4, 12.6);<br>2.85, *dd* (16.6, 2.9)
5	6.80, *d* (7.5)	6.80, *d* (8.0)
6	7.47, *dd* (8.3, 7.5)	7.47, *t* (8.0)
7	6.85, *d* (8.3)	6.84, *d* (8.0)
8-OH	-	10.80, *s*
1’	0.98, *d* (6.7)	0.88, *d* (6.6)
2’	0.94, *d* (6.4)	0.83, *d* (6.5)
3’	1.70, *m*	1.61, *m*
4’	1.82, *ddd* (13.9, 11.1, 4.2);<br>1.44, *ddd* (13.7, 9.8, 3.9)	1.67, *ddd* (13.4, 11.0, 4.4);<br>1.31, *ddd* (13.3, 9.5, 3.9)
5’	4.35, *m*	4.18, *m*
6’-NH	-	7.78, *m*
8’	4.19, *d* (5.9)	3.97, *d* (6.5)
8’-OH	-	5.94, *brs*
9’	4.03, *dd* (5.9, 4.0)	3.86, *d* (2.3)
9’-OH	-	5.54, *d* (5.0)
10’	3.73, *dt* (10.2, 3.7)	3.49, *m*
10’-NH_2	-	7.78, *m*
11’	2.93, *dd* (17.0, 3.3);<br>2.66, *dd* (17.0, 10.2)	2.65, *dd* (17.1, 2.9);<br>2.36, *dd* (17.0, 10.0)

12'-NH_2		7.61, *s*

Figure S8) LC-MS Chromatograms: TIC (top) and UV_{254} (bottom) for isolated **amicoumacin A.**

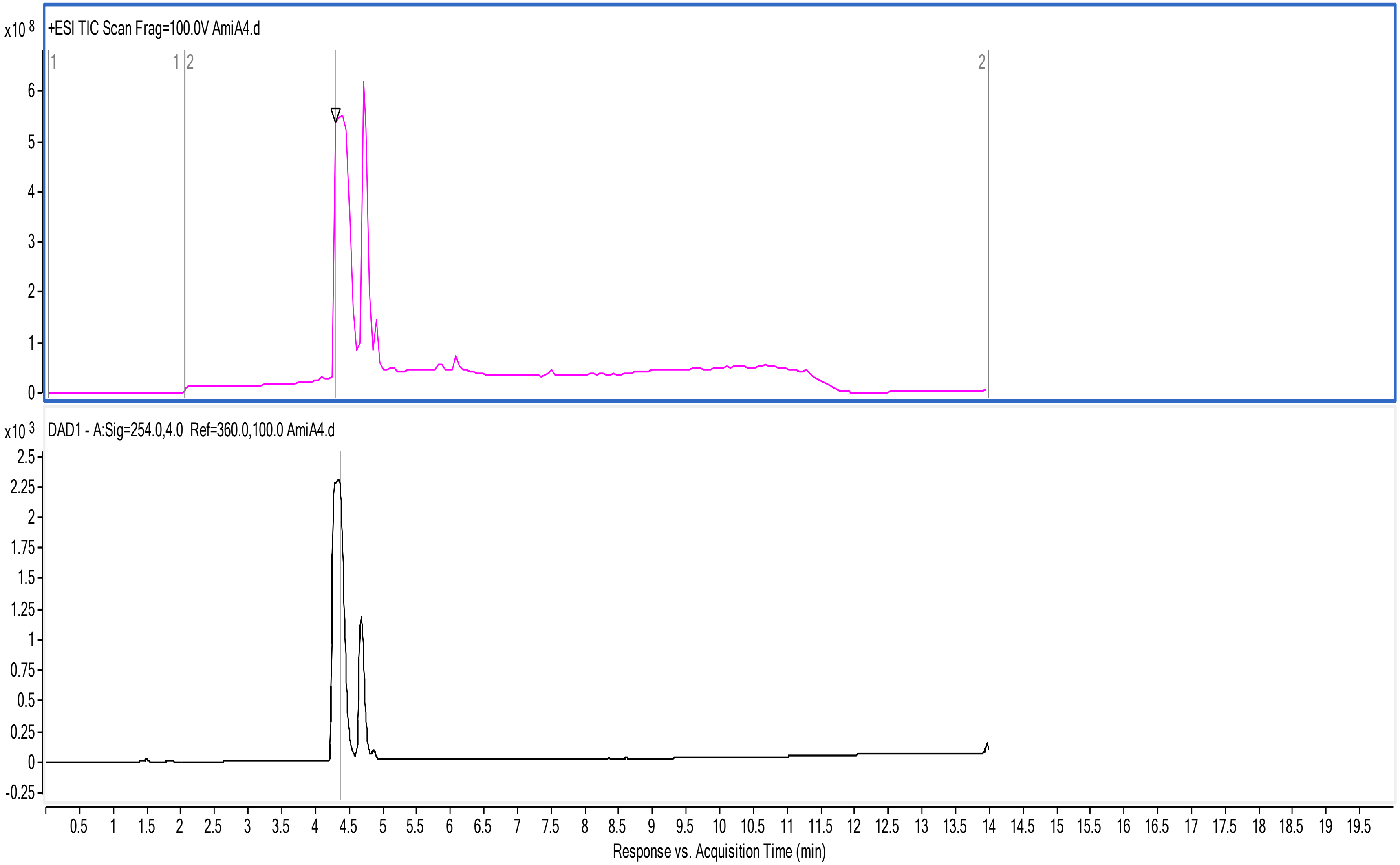

Figure S9) Mass spectrum (MS) for isolated **amicoumacin A.**

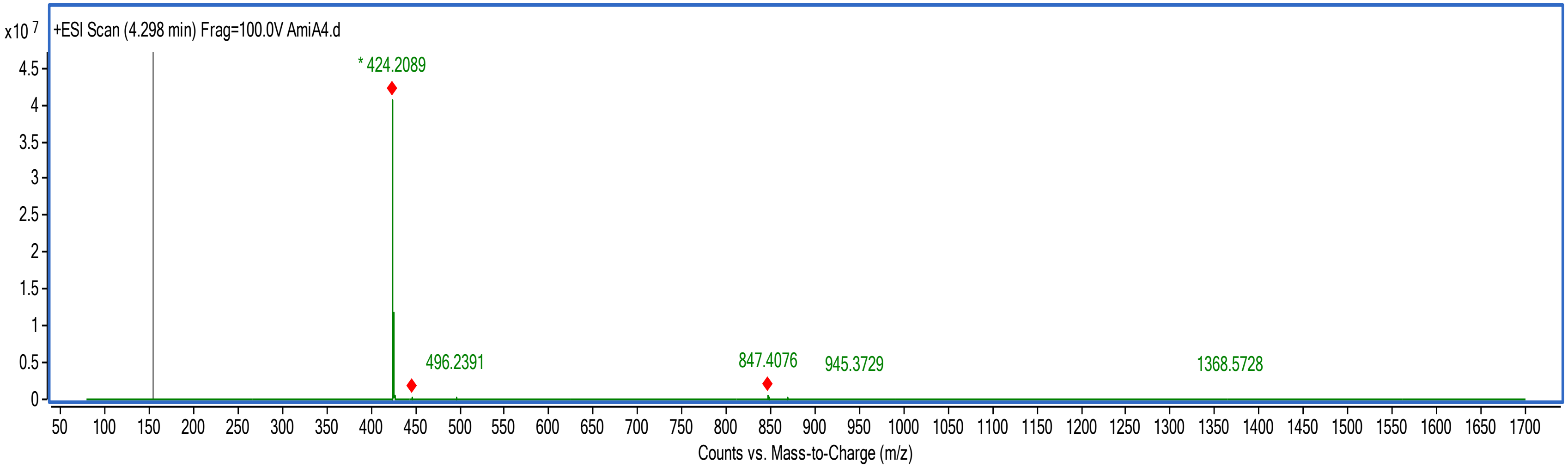

Figure S10) ^{1}H NMR spectrum (400 MHz, CD_3OD) for isolated **amicoumacin A.**

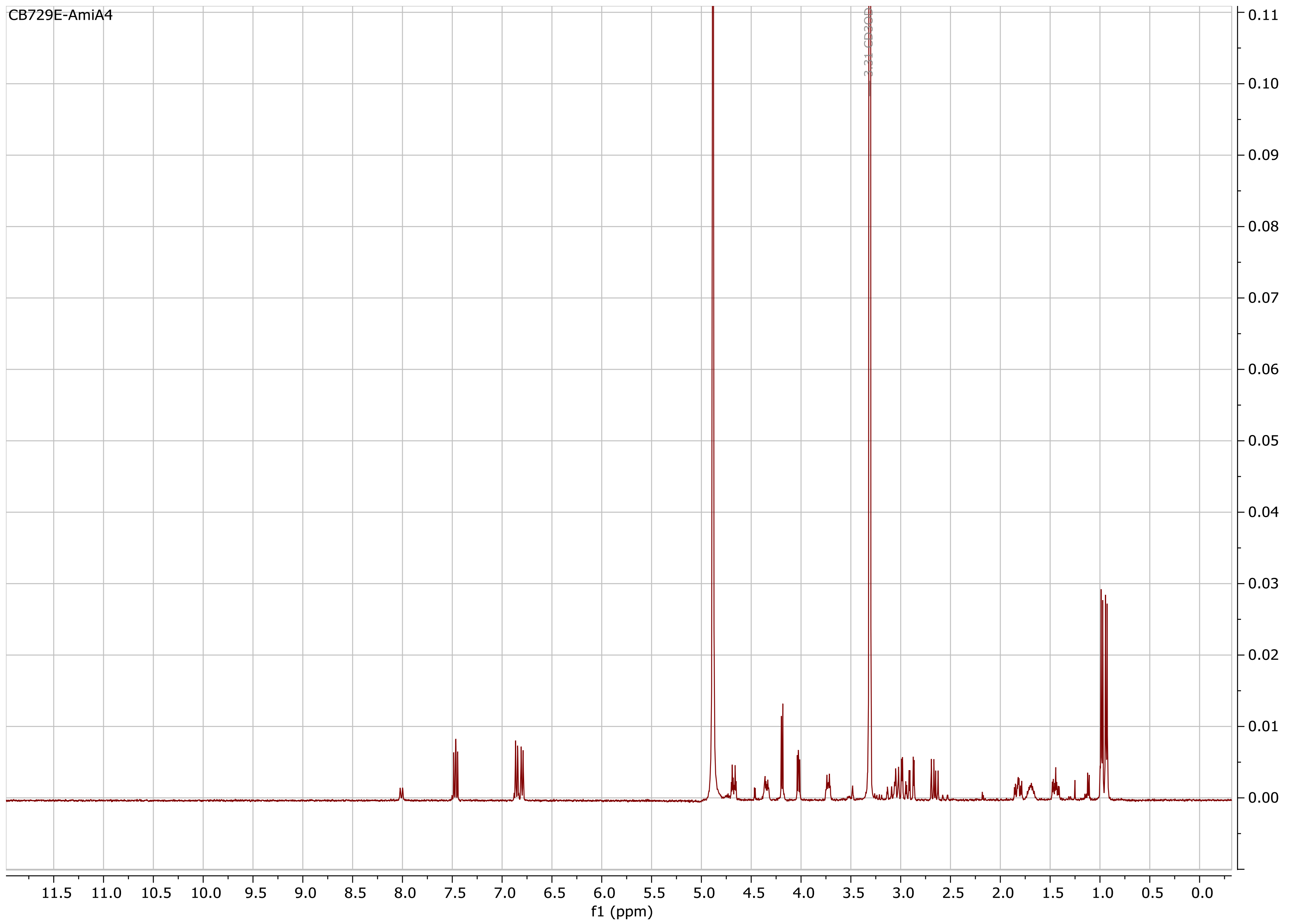

129

Table S5) ^{1}H and ^{13}C NMR chemical shifts of **amicoumacin C** in DMSO-d_6 as the major decomposition product of sample of purified amicoumacin A.

	Measured in DMSO-d_6	
position	δ_C, type	δ_H, mult, (J in Hz)
1	168.9	-
3	80.6, CH	4.72[a]
4	29.1, CH_2	2.97[a]
5	118.6, CH	6.84, *d* (7.8)
6	136.4, CH	7.50, *t* (8.0)
7	115.4, CH	6.87, *d* (8.5)
8	160.8, C	-
8-OH	-	10.81, *br s*
9	108.5, C	-
10	140.3, C	-
1’	23.4, CH_3	0.90, *d* (6.5)
2’	21.5, CH_3	0.83, *d* (6.5)
3’	24.0, CH	1.60[a]
4’	38.7, CH_2	1.68, *m*<br>1.34, *ddd* (13.4, 9.4, 4.1)
5’	48.6, CH	4.14, *ddd* (14.1, 9.5, 4.0)
5’-NH	-	8.01, *d* (9.4)
7’	170.0, C	-
8’	71.2, CH	4.33, *dd* (5.8, 3.4)
8’-OH	-	6.51, *d* (5.9)
9’	83.2, CH	4.69[a]
10’	47.6, CH	4.06, *br d* (8.7)
11’	33.6, CH_2	3.00[a]<br>2.48[b]

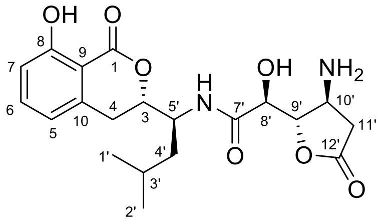

12’	173.9, C	-

[a] Overlapped with other signals.
[b] Overlapped with the solvent peak at 2.50.

Figure S11) LC-MS data of the decomposed sample of amicoumacin A, showing **amicoumacin C** as the major component: Total Absorbance Chromatogram (top), Total Ion Chromatogram (TIC, middle), and mass spectrum (bottom)

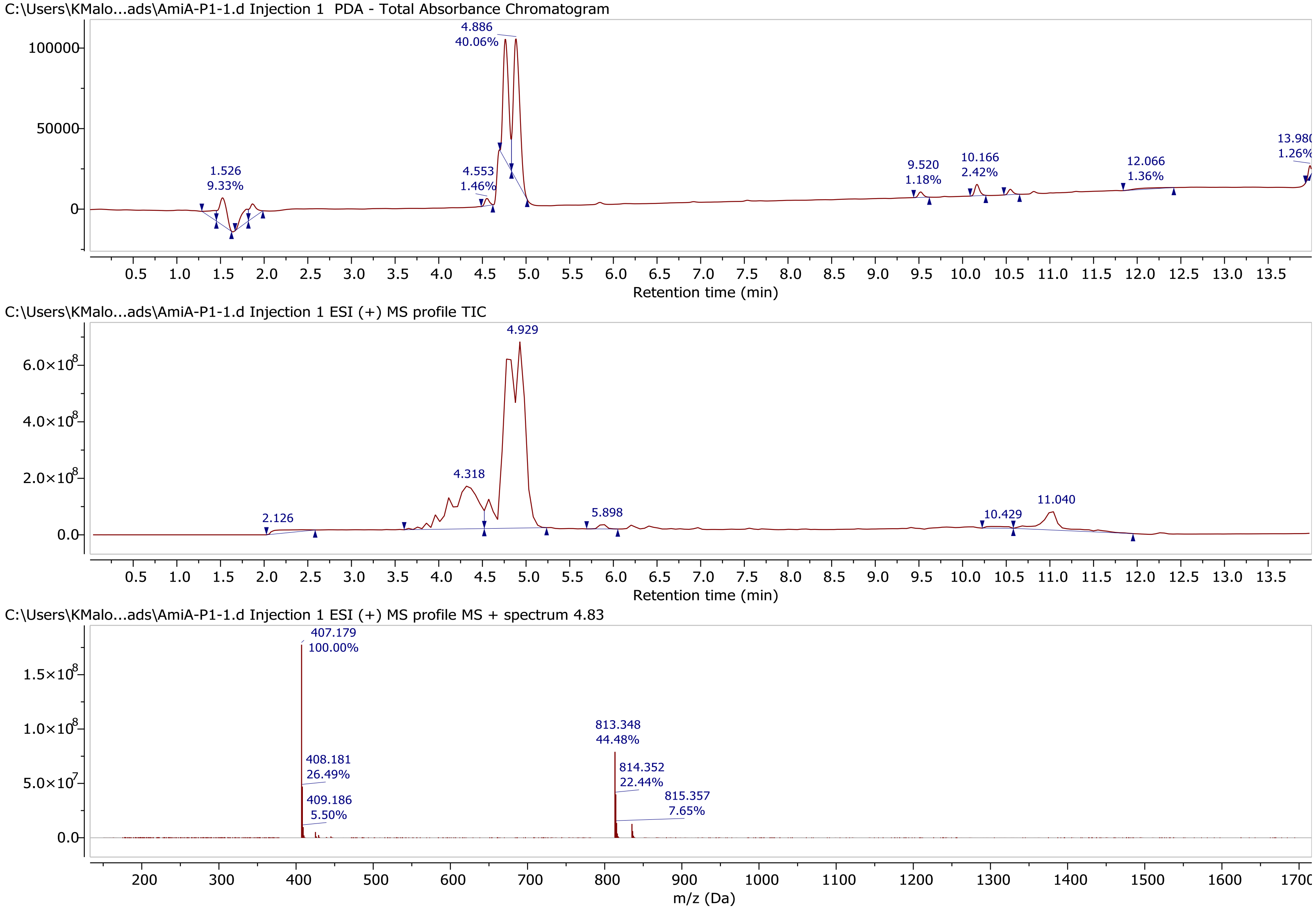

176 Figure S12) ^{1}H NMR spectrum (400 MHz, DMSO-d_6) for isolated **amicoumacin C** as the major decomposition product of a sample of purified
177 amicoumacin A.

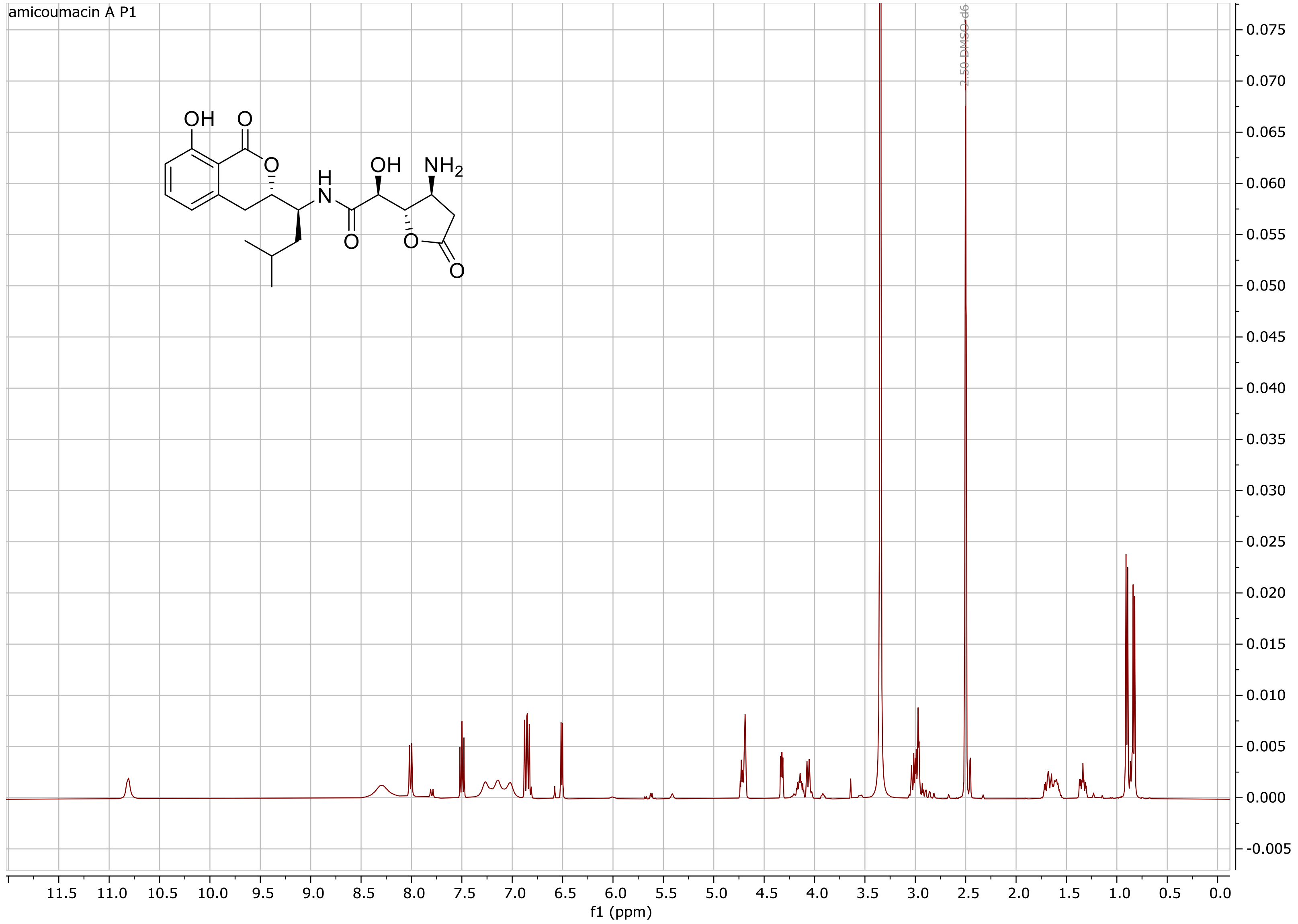

178

Figure S13) ^{13}C NMR spectrum (100 MHz, DMSO-d_6) for isolated **amicoumacin C** as the major decomposition product of a sample of purified amicoumacin A .

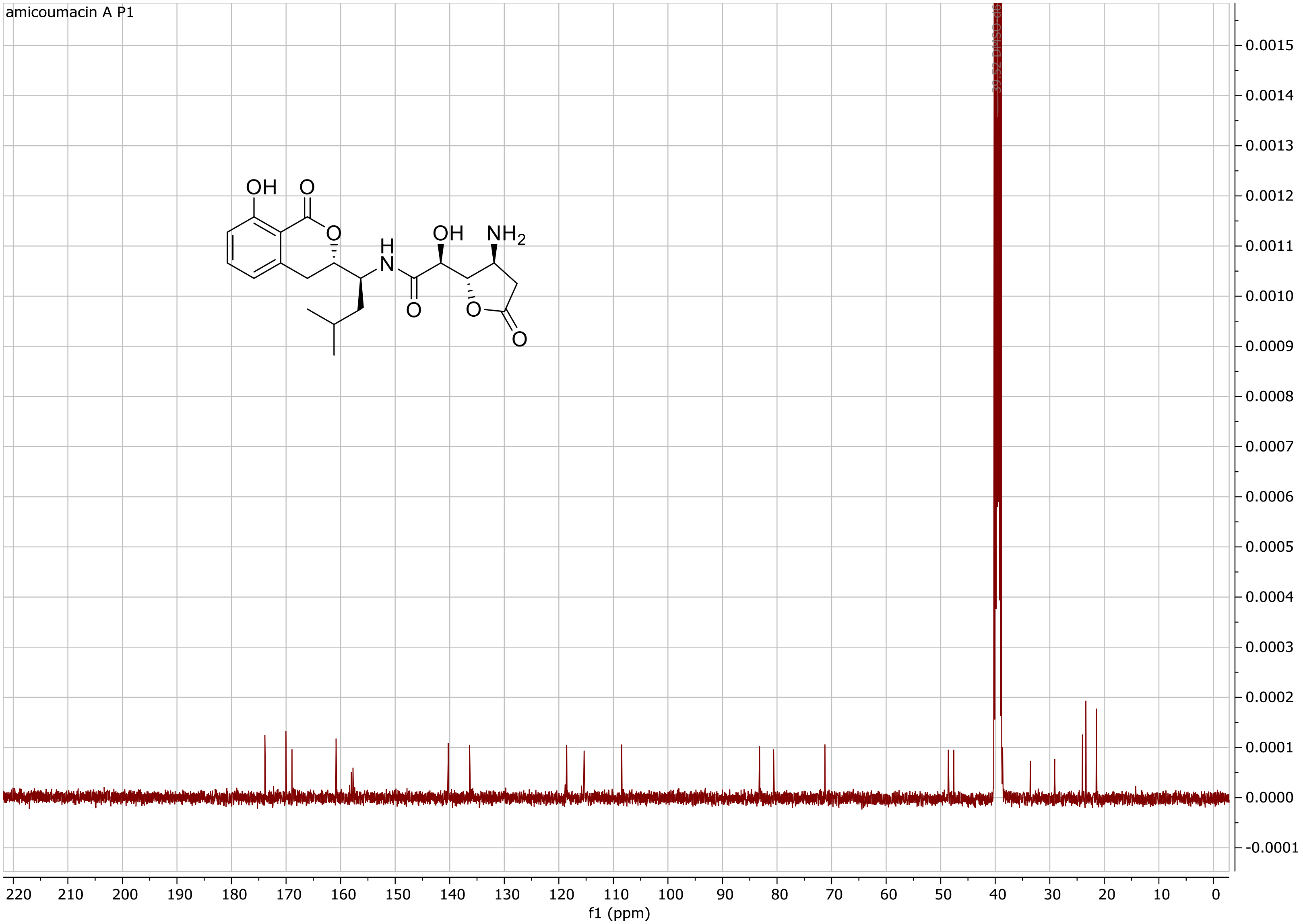

Figure S14) ^{1}H-13C HMBC spectrum (400 MHz, DMSO-d_6) for isolated **amicoumacin C** as the major decomposition product of a sample of purified amicoumacin A.

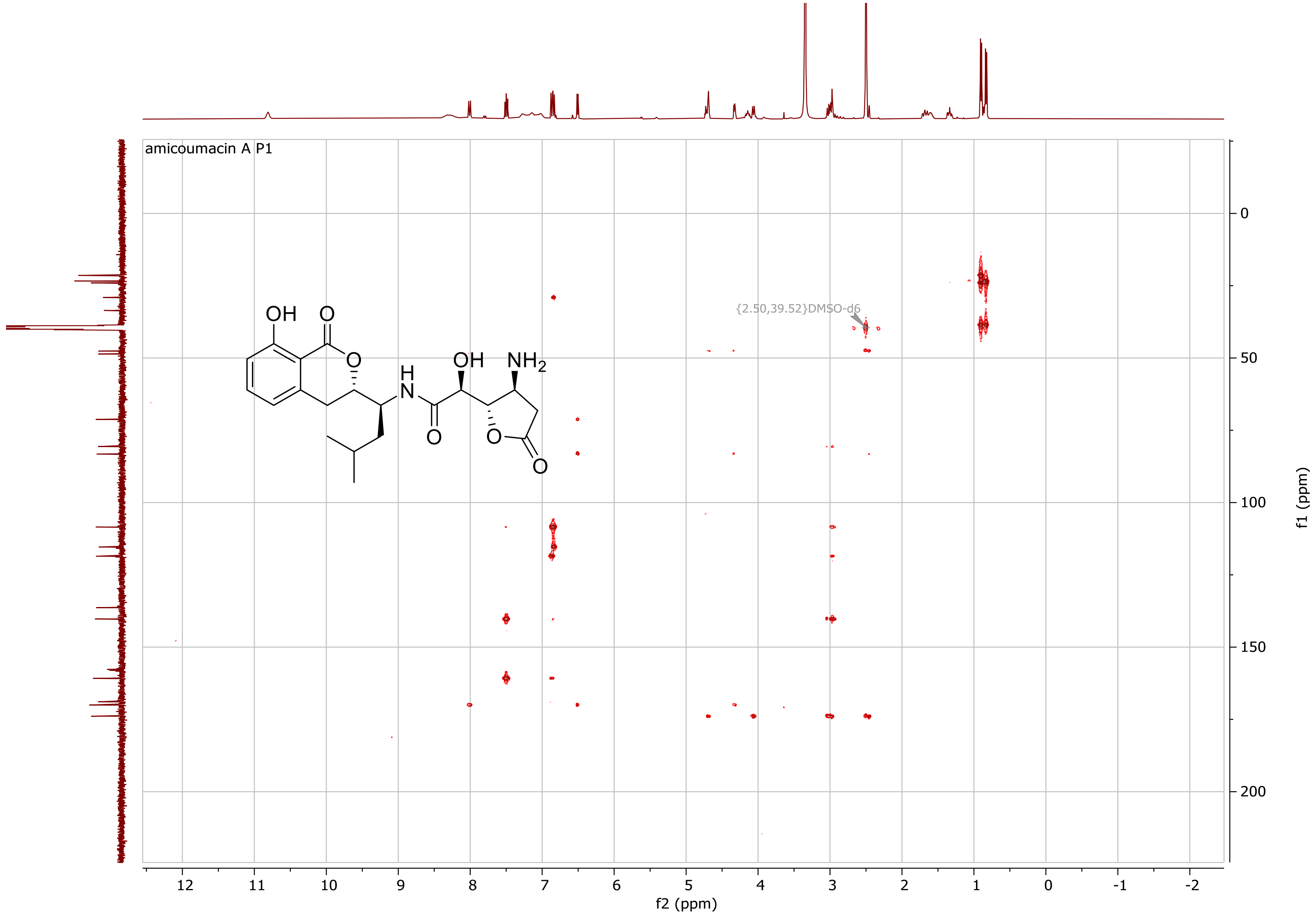

Figure S15) ^{1}H-13C HMBC spectrum (400 MHz, DMSO-d_6) for isolated **amicoumacin C** as the major decomposition product of a sample of purified amicoumacin A, zoomed in to show the correlation from H-9’ to C-12’.

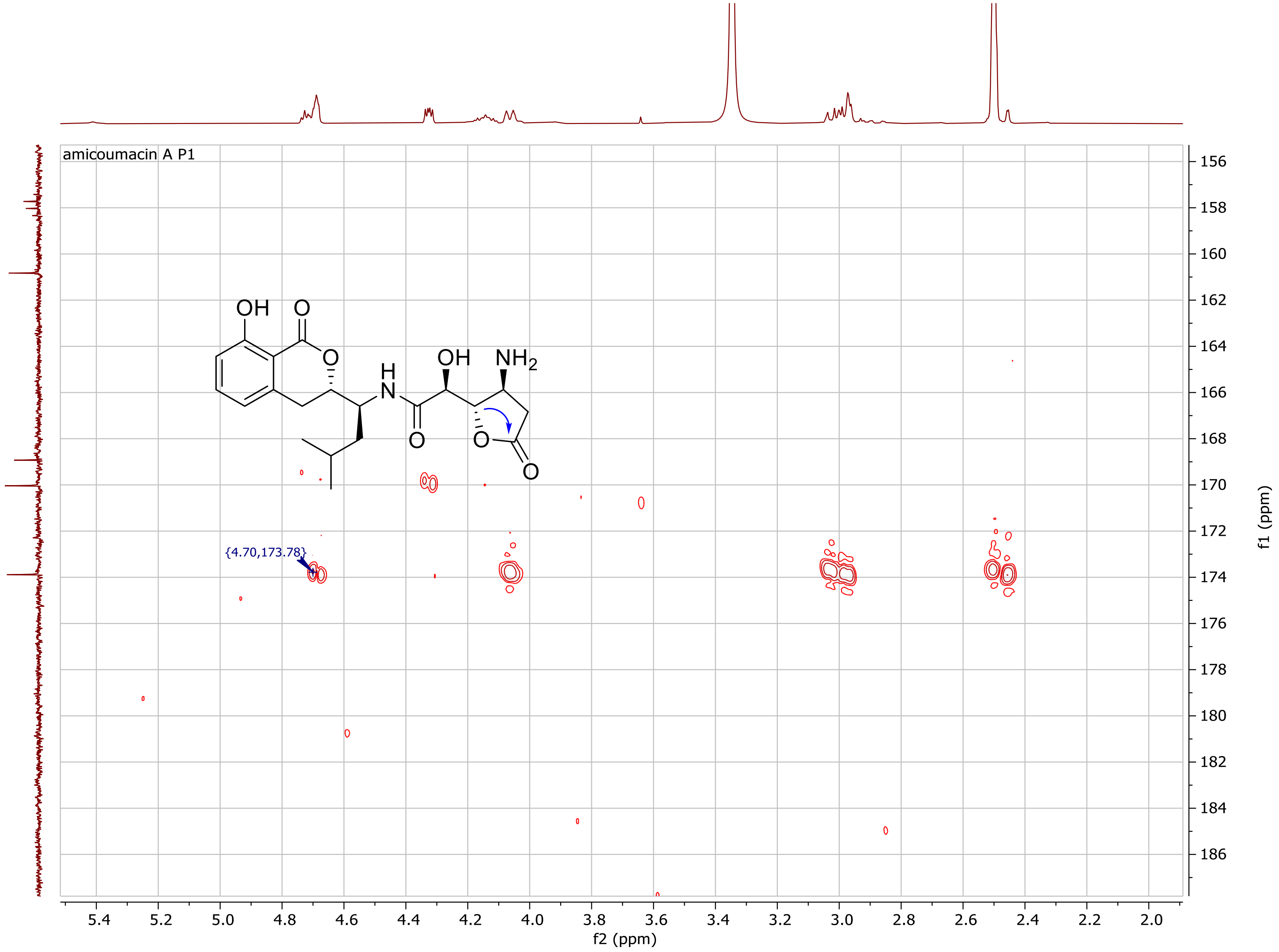

Figure S16 - (A) Extracted ion chromatogram of synthetic **amicoumacin A**. (B) MS2 compound analysis of synthetic **amicoumacin A**.

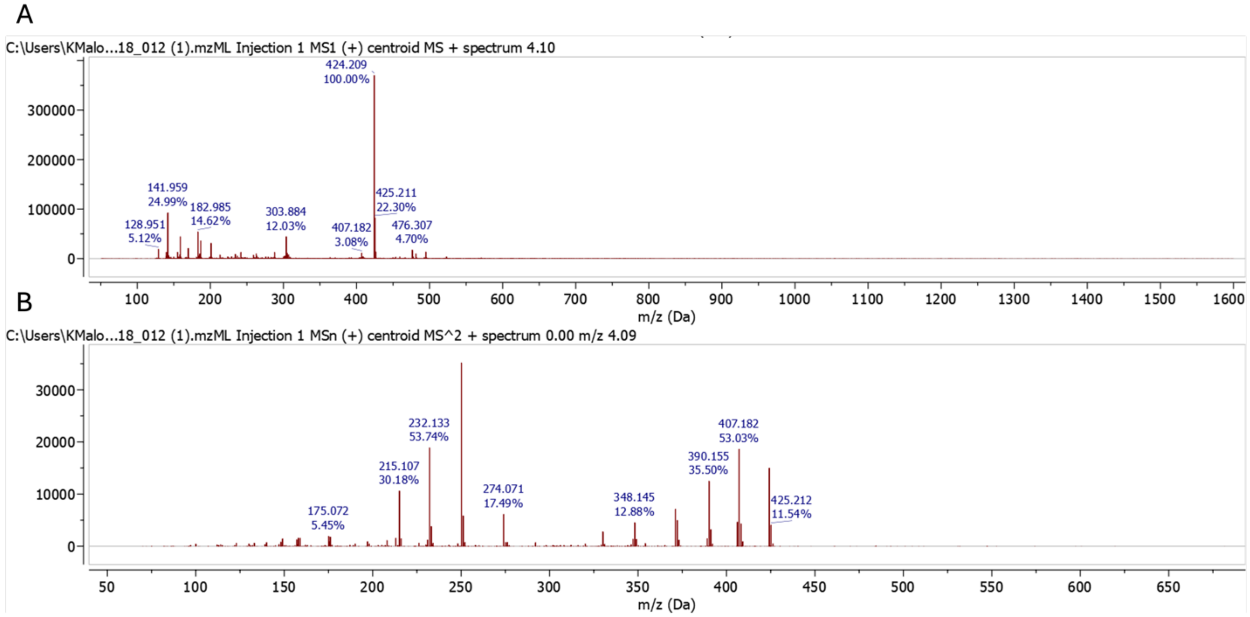

Figure S17 - (A) Extracted ion chromatogram of synthetic **amicoumacin B**. (B) MS2 compound analysis of synthetic **amicoumacin B**.

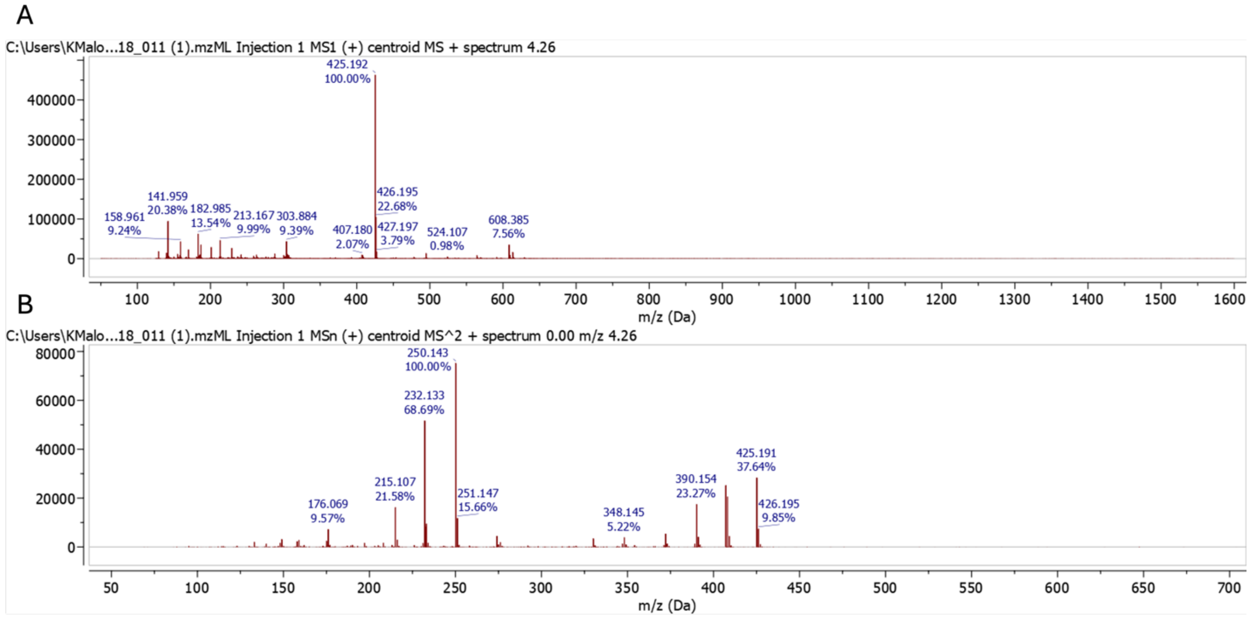

Supplement: Supplemental material — Tables S1 to S5; Figures S1 to S17. [file aem.00869-25-s0001.pdf]
